# Is Implicit Motor Learning Preserved after Stroke? A Systematic Review with Meta-Analysis

**DOI:** 10.1371/journal.pone.0166376

**Published:** 2016-12-16

**Authors:** E. Kal, M. Winters, J. van der Kamp, H. Houdijk, E. Groet, C. van Bennekom, E. Scherder

**Affiliations:** 1 Department of Research & Development, Heliomare Rehabilitation Centre, Wijk aan Zee, The Netherlands; 2 Department of Human Movement Sciences, Faculty of Behavioural and Movement Sciences, VU University Amsterdam, MOVE Research Institute, Amsterdam, The Netherlands; 3 Department of Clinical Neuropsychology, Faculty of Behavioural and Movement Sciences, VU University Amsterdam, Amsterdam, The Netherlands; 4 Department of Rehabilitation, Nursing Science & Sports, University Medical Centre Utrecht, Utrecht, The Netherlands; 5 Institute of Human Performance, University of Hong Kong, Hong Kong, Hong Kong; 6 Coronel Institute for Occupational and Environmental Health, Academic Medical Centre/University of Amsterdam, Amsterdam, The Netherlands; "INSERM", FRANCE

## Abstract

Many stroke patients experience difficulty with performing dual-tasks. A promising intervention to target this issue is implicit motor learning, as it should enhance patients’ automaticity of movement. Yet, although it is often thought that implicit motor learning is preserved post-stroke, evidence for this claim has not been systematically analysed yet. Therefore, we systematically reviewed whether implicit motor learning is preserved post-stroke, and whether patients benefit more from implicit than from explicit motor learning. We comprehensively searched conventional (MEDLINE, Cochrane, Embase, PEDro, PsycINFO) and grey literature databases (BIOSIS, Web of Science, OpenGrey, British Library, trial registries) for relevant reports. Two independent reviewers screened reports, extracted data, and performed a risk of bias assessment. Overall, we included 20 out of the 2177 identified reports that allow for a succinct evaluation of implicit motor learning. Of these, only 1 study investigated learning on a relatively complex, whole-body (balance board) task. All 19 other studies concerned variants of the serial-reaction time paradigm, with most of these focusing on learning with the unaffected hand (N = 13) rather than the affected hand or both hands (both: N = 4). Four of the 20 studies compared explicit and implicit motor learning post-stroke. Meta-analyses suggest that patients with stroke can learn implicitly with their unaffected side (mean difference (MD) = 69 ms, 95% CI[45.1, 92.9], *p <* .00001), but not with their affected side (standardized MD = -.11, 95% CI[-.45, .25], *p* = .56). Finally, implicit motor learning seemed equally effective as explicit motor learning post-stroke (SMD = -.54, 95% CI[-1.37, .29], *p* = .20). However, overall, the high risk of bias, small samples, and limited clinical relevance of most studies make it impossible to draw reliable conclusions regarding the effect of implicit motor learning strategies post-stroke. High quality studies with larger samples are warranted to test implicit motor learning in clinically relevant contexts.

## 1. Introduction

Most people consider going out for a walk while conversing with a friend to be an enjoyable and relaxing activity. With a moderate pace and a pleasant conversation, the cognitive (talking) and motor (walking) tasks can normally be performed concurrently without much effort. For many patients with stroke, however, this is not the case, as they often find themselves struggling to perform such cognitive-motor dual-tasks. Although up to 80% of patients regains walking ability [1], both gait [2] and postural control [3] often remain highly susceptible to interference from the concurrent performance of a cognitive task. This is not merely inconvenient, but actually compromises patients’ mobility and safety. For example, the ability to maintain gait speed above 0.7 m/s is assumed necessary for safely crossing a street [4]. Yet, performing an additional cognitive task can reduce walking speed well below this value in people with stroke [5,6]. In addition, heightened dual-task interference also increases the risk of falling [7]. Significantly, however, current rehabilitation practice does not seem particularly effective at recuperating dual-task performance [2].

Developing interventions to target dual-task interference requires knowledge of the aetiology of patients’ dual-task impairment. In general, explanations revolve around the dual-task framework of Abernethy [8] and working memory (WM) model of Baddeley [9]. Basically, when dual-tasking, the “central executive” is considered responsible for dividing the available attentional resources between the two tasks. As long as there are sufficient attentional resources *and* the central executive appropriately allocates these resources, no interference occurs. After stroke, however, WM-capacity is often reduced. For instance, slowed information processing as well as executive function deficits are commonly observed [10,11]. These deficits limit patients’ amount of attentional resources and their ability to appropriately allocate the resources between the tasks. In addition, many patients have difficulty with re-automating motor control, and use a highly cognitively-demanding strategy of consciously monitoring and controlling their movements [12,13]. As a result, motor tasks like walking may also place an increased *demand* on WM after stroke.

Based on the above, the two main ways to target dual-task interference post-stroke are (1) improving WM capacity and/or (2) reducing the WM demands associated with moving. Current evidence indicates that increasing WM-capacity is difficult, if not impossible (e.g., [14]). An alternative approach is to reduce WM load by (re-)automating motor control as much as possible, preferably in the initial phase of motor rehabilitation after stroke. Admittedly, it is unlikely that all patients will eventually attain the same level of automaticity as they had before they suffered brain damage. Still, we argue that patients' dual-tasking performance could already benefit from motor learning interventions that do result in some improvement in automaticity of movement. One intervention that seems especially fit for this purpose is implicit motor learning. In the current paper, we will systematically review evidence to determine whether this mode of motor learning is actually preserved in people with stroke. First though, we will shortly introduce the concept of implicit motor learning, and explain why it might be a promising intervention to improve motor functioning and dual-tasking post-stroke.

### 1.1 Different routes to movement automaticity after stroke: Explicit and implicit motor learning

**Fig 1:**
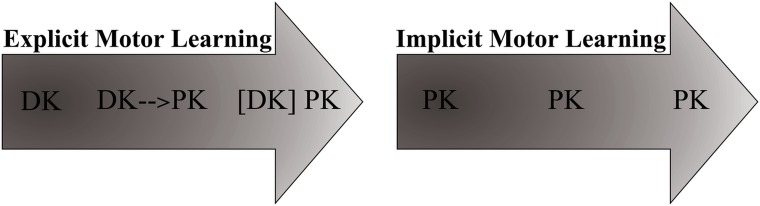
Knowledge types underlying motor control throughout explicit and implicit motor learning [15,16]. With explicit motor learning, motor control first relies on declarative knowledge (DK), which in the course of practice is gradually transformed into procedural form (PK). Although no longer essential for motor control, declarative knowledge remains accessible in the automatic phase ([DK]). In contrast, during implicit learning, motor control depends on procedural knowledge right from the outset of learning, with practice resulting in more refined procedural knowledge. Thus, although both learning modes eventually result in fully procedural motor control, only explicit learning results in the accrual of declarative knowledge. Please note that this dichotomous model is a simplified representation of motor learning. Learning is likely to involve both modes of learning in parallel or in interaction, and is not either purely implicit or purely explicit [19–21].

Traditional views on skill acquisition [15,16] hold that in the early ‘verbal-cognitive’ phase of motor learning, motor control requires considerable WM involvement; novices accrue and employ declarative movement-related rules and strategies to consciously control motor performance. In the course of learning, however, motor control becomes progressively less dependent on declarative knowledge and instead increasingly relies on procedural knowledge that directly links task-relevant information to the desired motor response [15]. Since procedural knowledge does not require conscious processing, motor control becomes less dependent on working memory contributions. After extensive practice, finally, the ‘automatic phase’ is reached, in which motor control has become fully procedural. This view on motor learning–involving a shift from declarative toward procedural control of movement–is typically called *explicit* motor learning [17] (see Fig 1). Specifically, according to consensus among experts explicit motor learning is: “… learning which generates verbal knowledge of movement performance (e.g. facts and rules), involves cognitive stages within the learning process and is dependent on working memory involvement” ([18], p.5).

Observational studies of current stroke rehabilitation practice show that physical therapists often rely on these explicit motor learning strategies: mainly providing verbal instructions and feedback concerning *how* movements should be performed, thereby eliciting conscious attempts on the part of the patients to adjust motor performance [22,23]. Theoretically, this apparent bias toward using explicit motor learning strategies should not be a cause of concern, as this mode of learning can eventually result in fully automated motor performance. However, in practice, many patients remain strongly inclined to consciously control their movements (and in a way, remain “stuck” in the verbal-cognitive phase) up to years after discharge [12,13,24]. For these patients, motor control remains highly WM-dependent and, hence, susceptible to dual-task interference.

One way to diminish this problem might be *implicit motor learning*. In contrast to explicit motor learning, implicit motor learning “… progresses with no or minimal increase in verbal knowledge of movement performance (e.g., facts and rules) and without awareness. Implicitly learned skills are (unconsciously) retrieved from implicit memory.” ([18], p.6) In other words, when learning a movement implicitly, the learner largely skips the declarative phase of learning and hence acquires far less explicit movement-related knowledge. Instead, the learner directly develops procedural knowledge of the skill instead (Fig 1). As a result, implicit motor learning requires no or minimal conscious involvement, and only minimally loads WM. Hence, movements should be less easily disturbed by dual-task performance.

A typical example of implicit motor learning is unintentional learning, such as in the serial-reaction time (or SRT) task. For this task, participants are presented with a sequence of visual stimuli, appearing at different locations on a computer screen. Participants are required to press the button that corresponds with this location as fast as possible. Unbeknownst to the participants, stimuli are not randomly presented but follow an embedded repeating sequence. After practice, implicit motor learning is evidenced by the fact that participants generally respond significantly faster on these sequenced stimuli than on randomly presented ones, without being able to explicitly recall or recognize this learned sequence [25,26].

Motor skills with more complex movement dynamics (e.g., balancing) can also be learned implicitly. Compared to SRT tasks, it is more difficult to learn such complex motor tasks in a purely implicit way–when learning to stabilize a balance board, learners will likely always have some explicit knowledge of how to perform the task. Nonetheless, there are implicit motor learning methods available that can minimize the involvement of such explicit processes. Although there is some debate as to the most effective method, the following three are most often used and generally agreed upon to yield reliable results [18]: 1) dual-task learning: performing an attention demanding secondary task during motor learning, which consumes large proportions of WM capacity and hence impairs the learner’s ability to process movement-related knowledge [17,27,28]. A typical example is a study by Maxwell et al. [27] in which participants implicitly learned a golf-putting task by simultaneously counting tones that were presented every 1–2 seconds; 2) analogy learning: providing the learner with a metaphor that encompasses the global structure of the to-be-learned skill, such that only minimal WM involvement is required [29,30]. For example, when learning a table-tennis forehand stroke, an effective analogy is to “move the bat as though it is travelling up the side of a mountain” (Koedijker et al., *p*. 251; [29]); and 3) errorless learning: constraining the learning environment to ensure that very few errors occur and learners are not enticed to engage in (WM-demanding) hypothesis-testing behavior [31,32]. In the study by Lam et al. [32], for instance, the occurrence of errors was minimized by having participants first put from a very short distance (0.25 cm), which was subsequently only gradually increased. Finally, although not always earmarked as such, learning using an external focus of attention (i.e., focusing attention on movement effects) may induce implicit motor skill learning, as it minimally taxes WM [33,34] and results in the accrual of limited amounts of movement-related declarative knowledge [35]–the hallmark of implicit motor learning. For instance, when taking a step, focusing externally on *where* to place your feet has been found to result in more automatic movement execution compared to focusing internally on the stepping movement itself [33].

Within healthy adults, the paradigms outlined above have generated convincing evidence for the WM-independence of implicit motor learning. For instance, implicit motor learning seems less reliant on neural networks involved in executive WM control (i.e., prefrontal and premotor cortices [36–40]) than explicit motor learning. Also, a learner’s WM capacity is not associated with the rate of implicit motor learning, while it does predict the rate of explicit learning (see Janacsek & Nemeth for a review [25]), and age-related reductions in WM capacity primarily affect explicit, not implicit, motor learning abilities [41,42]. Finally, and most importantly, numerous studies show that—compared to explicitly learned movements—the performance of implicitly acquired motor skills is more robust to concurrent performance of a wide variety of cognitive tasks. Examples include: tone-counting during golf-putting [35,43], random-letter generation during surgical knot-tying [44], number-recall during balancing [45], and word-monitoring during table-tennis forehand strokes [29,46].

Considering the promising findings within healthy adults, one would hypothesize that dual-task performance of patients with stroke can be enhanced through the use of implicit motor learning strategies during rehabilitation. However, one vital precondition must be met for this conjecture to be true, namely that patients actually retain the ability to learn implicitly after stroke. Problematically though, it is not yet clear whether and to what degree this is the case. Although several studies have reported implicit motor learning to be preserved post-stroke [47–51], others have reported that implicit motor learning to be impaired or even absent [52–55].

Therefore, in order to determine the suitability of implicit motor learning as an intervention during rehabilitation post-stroke, our current aim is to assess whether implicit motor learning is still possible after stroke. To this end, we will systematically review studies that have investigated implicit motor learning after stroke, focusing on the following research questions: 1) Can patients with stroke learn motor tasks implicitly–i.e., improve their motor skill, without the accrual of declarative movement-related knowledge? 2) Is implicit motor learning impaired in patients compared to healthy peers? 3) Is implicit motor learning more or less impaired than explicit motor learning following stroke?

## 2. Methods

### 2.1 Criteria for inclusion of studies

The following in- and exclusion criteria were applied in the selection of papers.

#### 2.1.1 Population

Only studies that concerned patients with stroke were included (>18 years of age). Studies were excluded if patient groups were mixed in terms of lesion etiology (i.e., not only stroke), unless implicit motor learning could be assessed separately for the stroke group. If studies were based on the same patient cohort, only the data from the first published study was included.

#### 2.1.2 Experimental design

Published and non-published studies that investigated implicit *motor* learning were included. Both randomized and non-randomized (i.e., quasi-randomized, controlled before-and-after studies, cohort studies, case-control studies) studies that assessed motor learning with immediate or delayed retention tests were eligible for inclusion. Case studies were excluded. Further, we only included studies that checked whether patients did not acquire explicit movement-related knowledge in the course of learning (i.e., by means of verbal reports, recognition/recall tests, or awareness tests). This because without such checks it cannot be ascertained that motor learning had indeed been implicit. As this review did not aim to assess the effect of an intervention (i.e., brain stimulation or medication) on implicit motor learning post-stroke, intervention studies were included only if they also assessed implicit motor learning within a non-exposed (i.e., placebo or control) patient group.

#### 2.1.3 Assessment of motor learning

Studies that used (versions of) SRT paradigms were eligible for inclusion if the difference in tracking error/reaction time between random and repeated motor sequences could be obtained [56,57]. Studies that investigated learning on more complex motor tasks (i.e., balancing, grasping, walking) were included if they assessed performance improvement from baseline to post-test.

### 2.2 Data sources and searches

#### 2.2.1 Database search

We searched the following databases (from inception to 1 October 2015) for relevant studies: MEDLINE, the Cochrane library, Embase, PEDro, and PsycINFO. A medical research librarian developed a sensitive search strategy, using controlled vocabulary and free text search terms. We did not impose any language restrictions. The search strategy can be divided in the following key parts: Implicit (#1), Learning (#2), Memory (#3), Motor Performance (#4), and Brain Injury (#5). These terms were adapted to each database's terminology, and if applicable, the so-called explode feature was used to search for more specific related terms. For each database, the key search features were combined in the following fashion: (#1 AND (#2 OR #3)) AND #4 AND #5. S1 Text lists the MEDLINE search strategy.

#### 2.2.2 Grey literature and ongoing studies

Unpublished reports and conference abstracts were searched for in BIOSIS Previews, Web of Science, OpenGrey, and the British Library. To identify possibly relevant ongoing studies, national (http://www.trialregister.nl) and international trial registers (https://clinicaltrials.gov; http://apps.who.int/trialsearch/) were searched. When a possibly relevant ongoing study was found, its primary investigator was contacted to acquire further information on the study.

#### 2.2.3 Hand searching

Reference lists of included studies and relevant reviews were screened for additional relevant studies.

### 2.3 Study selection

After removal of duplicates, two reviewers (EK, JvdK) independently examined titles and abstracts of all identified studies to determine their eligibility. Next, the two reviewers independently examined the full text of these studies, and applied the in- and exclusion criteria to determine their eligibility. If discrepancies existed, reviewers conferred to reach consensus on this issue. A third independent reviewer (HH) was consulted if no consensus could be reached.

### 2.4 Data extraction and quality assessment

The two reviewers independently extracted the following information from the included studies:

- Study population (number of participants, age, gender, time since stroke, stroke location, results of tests of cognitive and motor functioning);
- Study characteristics (type of motor task, content of training, retention on separate day (yes/no), declarative knowledge tests and their results);
- Study results: for dynamically complex motor tasks: performance improvement from pre- to post-test; For SRT-type paradigms: difference in performance on random vs. sequenced stimuli;

The two reviewers independently assessed the risk of bias of the included studies with the Newcastle-Ottawa Scale (NOS [58]), which was slightly modified for the study purpose (as recommended by the Cochrane Handbook [59]; see Appendices A-C in S2 Text). Three separate versions of the NOS were used. The first NOS was used to rate studies’ quality to answer the main research question (Can patients with stroke learn motor tasks implicitly?). The scale contains items on participant selection, performance bias, and outcome assessment, with scores ranging from 0–8 (Appendix A in S2 Text). The second and third NOS (Appendices B and C in S2 Text) were used to rate studies’ risk of bias regarding the sub questions: “Is implicit motor learning impaired after stroke compared to healthy peers?” and “Is implicit motor learning more or less impaired than explicit motor learning following stroke?”. These NOS scales contained the same items as the first NOS, plus items regarding group comparability. Scores could range between 0–12. Higher NOS-scores reflect a lower risk of bias. In this review, studies could either be classified as exhibiting a high (NOS-1: 0–4; NOS-2&3: 0–8), moderate (NOS-1: 5–6; NOS-2&3: 9–10), or low risk of bias (NOS-1: 7–8; NOS-2&3: 11–12).

### 2.5 Data synthesis and analysis

Data synthesis: Data pooling was carried out with RevMan 5.3 (The Nordic Cochrane Centre, Copenhagen, Denmark) by two authors (EK/MW). We planned analyses for all three research questions. Based on clinical grounds, we a priori decided to only pool data when similar task paradigms and motor effectors were used (e.g., lower/upper limb; affected/unaffected side/bilateral involvement). From a clinical point of view, this distinction is relevant, as rehabilitation practice is primarily concerned with restoring motor function of the patient’s affected side, rather than the unaffected side. In addition, from a theoretical point of view, this approach also allowed us to assess whether stroke patients suffer from general, effector-independent implicit learning deficits (i.e., a general deficit in sequencing each sub-movement of the motor skill, regardless of the extremity involved), and/or from effector-dependent impairments (i.e., a specific deficit in learning the performance of each sub-movement using the most-affected extremity; see [60–62]).

When studies used the same outcome measure (with similar units of measurement) data were pooled using the mean difference (MD). For studies that used different outcome measures we used the standardized MD (SMD; i.e., Cohen’s *d* corrected for bias in studies with small samples [63]). Significance level was set at *p* < 0.05. A fixed effects model was used to pool data when studies were statistically homogenous, or when fewer than 5 studies were available for data synthesis. A random effects model was only used when both heterogeneity was present and when more than 5 studies were available. Statistical heterogeneity was assessed by visually inspecting the forest plots, and by means of the I^2^-statistic, with heterogeneity being present when the X^2^ was significant (*p* < 0.1) [59]. Causes of statistical heterogeneity were further explored with meta-regression or subgroup analyses, if appropriate (i.e., ≥ 10 studies available for synthesis). With regard to the latter, we specifically planned subgroup analyses to explore whether statistical heterogeneity was due to between-study differences between studies in patients’ lesion location. For this purpose, we classified the lesion location of patients in each study (i.e., cortical, subcortical, mixed cortical/subcortical, cerebellar stroke; [64]). Descriptive synthesis was presented in case data pooling was not considered feasible. A funnel plot was used to investigate the presence of publication bias [65].

## 3. Results

### 3.1 Literature search

**Fig 2:**
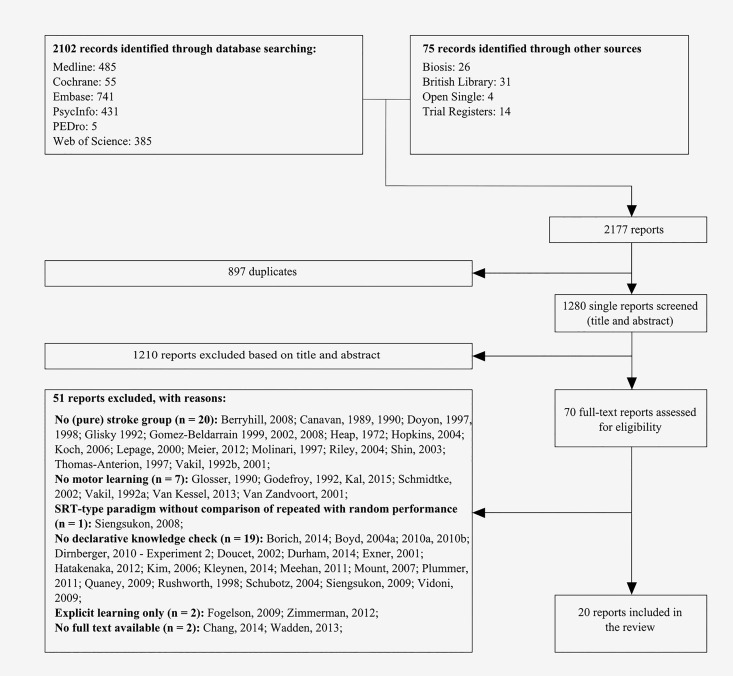
Flow-chart of inclusion of studies. Note that only experiment 1 of the study of Dirnberger et al. [74] was included, while experiment 2 was excluded. Therefore, although 51 out of the available 70 reports were excluded after full-text screening, 20 reports were included.

In total, our search identified 2177 reports. After removal of duplicates and screening of titles and abstracts, full text reports were obtained for 70 studies. Application of the in- and exclusion criteria eventually resulted in the inclusion of 20 studies (see Fig 2). Most of the excluded studies included heterogenic patient groups (i.e., not only stroke; n = 20), or did not check whether learning had been implicit (n = 19). Despite successive attempts, no full text could be obtained for 2 studies [66,67]. Note that two of the included studies were written in Korean [68,69]. These were translated into English by a native Korean scientist with experience in the field of (implicit) motor learning. Inspection of a funnel plot of all included studies revealed no evidence of publication bias, considering its symmetrical distribution (See S1 Fig) [62,65].

### 3.2 Study characteristics

S1 Table summarizes the characteristics of the 20 included studies.

#### 3.2.1 Design and implicit motor learning paradigms

With the exception of two studies [52,70], all studies compared implicit motor learning abilities of patients with stroke with those of healthy age-matched controls. Four studies also contrasted the effectiveness of implicit motor learning and explicit motor learning after stroke [32,47,50,52]. Note that several studies incorporated multiple stroke patient groups [32,49,52,71,72]. With regards to the experimental paradigm used, almost all studies have focused on motor learning involving the upper extremity. Specifically, most studies (*N* = 14) investigated implicit motor learning using the SRT-paradigm.[47,49,51,52,54,55,68–70,73–77] Adapted versions of this SRT-paradigm were also used, either in the form of the so-called serial hand movement (SHM) paradigm (*N* = 2) [72,78] or continuous tracking (CTT) task (*N* = 2) [50,79]. Both these paradigms are essentially similar to the SRT, but require slightly more complex hand movements such as the handling of switches (SHM) or tracking of a continuously moving target with a hand-held stylus (CTT). In one study patients learned both the SRT and SHM task [71], to explore if task difficulty influences motor learning ability post stroke. Importantly, almost all of the above studies investigated implicit motor learning using the relatively unaffected upper extremity (*N* = 13)[47,49,50–52,54,68,69,71–73,77,78]. Four studies also investigated implicit motor learning using the affected upper extremity [51,54,70,79], whereas in four other studies motor performance required bilateral movements (i.e., the middle and index finger of each hand) [55,74–76]. Finally, only one study assessed implicit motor learning within the context of learning a dynamically more complex motor task–stabilizing a balance board using an errorless learning approach [32].

#### 3.2.2 Participants

In total, 337 patients and 253 controls participated in the selected studies. Across studies, considerable heterogeneity was noted in terms of patient characteristics, such as patients’ mean age (range: 46–74 years), time since stroke (range: 1.9–88 months) and lesion location. With regard to the latter, three studies investigated patients with isolated cerebellar lesions [54,74,75], six studies incorporated patient groups with isolated supratentatorial subcortical lesions [50,51,55,73,76,79], four studies studied patient groups with mixed supratentatorial subcortical- and/or cortical lesions [49,52,70,71], while 2 studies incorporated patient groups with mixed sub- and supratentatorial lesions [32,52]. Finally, in five studies lesion location was only described as being supratentatorial and not further specified [68,69,72,77,78].

### 3.3 Quality assessment

Table 1 shows the NOS-scores of each study. Overall, most studies exhibited moderate to high risk of bias (see the supplementary material for justification of NOS-scores). This was for a large part due to lack of detail on participant screening and selection (cf [70]), lack of assessment of/correction for confounding factors [47,49–51,55,68,69,71–73,75–79], and lack of reporting on the amount of participants’ explicit movement-related knowledge [49,51,54,68–70,72,78]. In fact, in some studies participants gained so much explicit knowledge that learning may have been explicit, rather than implicit [55,71,73,77].

**Table 1:** NOS-scores of included studies.

*Study*	NOS-1:	NOS-2:	NOS-3:
	Implicit motor learning in stroke (0–8)	Implicit motor learning in stroke vs. controls (0–12)	Implicit vs. explicit motor learning in stroke (0–12)
Boyd & Winstein, 2001 [52]	**5.5**		**6**
Boyd & Winstein, 2003 [47]	**7**	**9**	**11**
Boyd & Winstein, 2004 [50]	**7**	**9**	**10**
Boyd et al., 2007 [71]	**4**	**6**	
Boyd et al., 2009 [73]	**5**	**7**	
Dirnberger et al., 2010 –Experiment 1 [74]	**6**	**9**	
Dirnberger et al., 2013 [75]	**6**	**8**	
Dovern et al., 2011 [49]	**3**	**3**	
Exner et al., 2002 [76]	**5**	**6**	
Gomez et al., 1998 [54]	**2**	**3**	
Lee et al., 2006 [68]	**2**	**2**	
Lee et al., 2008 [69]	**1**	**1**	
Meehan et al., 2011 [79]	**6**	**8**	
Orrell et al., 2006 [32]	**6**	**9**	**10**
Orrell et al., 2007 [77]	**4**	**5**	
Pohl et al., 2001 [78]	**3**	**6**	
Pohl et al., 2006 [72]	**3**	**6**	
Rösser et al., 2008 [70]	**3**		
Shin et al., 2005 [51]	**1**	**2**	
Vakil et al., 2000 [55]	**3**	**5**	

NB: Scores are presented separately for each research question. Colours indicate overall risk of bias assessment, with darker grey indicating high risk of bias, grey indicating moderate risk of bias, and lighter grey representing low risk of bias.

### 3.4 Data synthesis

#### 3.4.1 Research question 1: Can patients with stroke learn motor tasks implicitly?

***3*.*4*.*1*.*1 SRT-Type tasks—Learning using the unaffected upper-extremity***. Of the thirteen studies that investigated implicit motor learning using the unaffected upper extremity, eleven were eligible for data pooling [47,49,51,52,54,68,69,71–73,78]. For one study no information on the variance of the learning effect could be obtained [77]. Therefore, this study is discussed in the descriptive synthesis section below, along with one study by Boyd and Winstein [50] which could also not be included in the meta-analysis. This because in this latter study a CTT paradigm was used to assess implicit motor learning, measuring the learning effect in percentage RMSE. This is in contrast to the other eleven SRT- and SHM-studies, which measured learning in milliseconds. Technically we could have pooled all twelve studies with SMDs. However, this would have violated the assumption that between-study variation in SDs is due to the use of different measurement scales rather than to differences in variability among study populations [59]. Therefore, we chose not to do this and only descriptively present Boyd and Winstein’s [50] findings.

**Fig 3:**
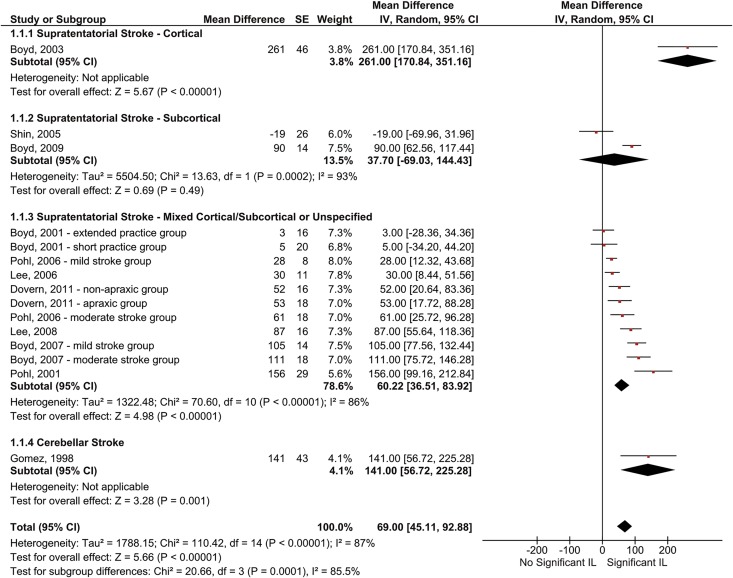
Pooled results of SRT-/SHM-studies that investigated implicit motor learning after stroke for the unaffected hand. Results concern the mean differences in reaction time (in ms) between repeated and random blocks. Square size indicates a study’s relative contribution to the pooled estimate. Diamond width indicates the 95% confidence interval of the pooled effect. Note that Boyd et al. [69] tested patients on both a SHM and a SRT paradigm. We therefore collapsed the data for each group across these paradigms, following Cochrane recommendations [59]. NB: CI = confidence interval; IL = implicit motor learning; IV = inverse variance; SE = standard error;

The eleven studies that were pooled incorporated 15 stroke groups. A random effects model was used with the mean difference in reaction time between random and repeated blocks serving as outcome measure. Results showed that patients demonstrated significant implicit motor learning with their unaffected hand, as evidenced by faster reaction times on the repeated compared to the random blocks (MD = 69 ms, 95% CI = [45.1, 92.9], Z = 5.66, *p* < .00001; Fig 3). Considerable statistical heterogeneity was present (*I*^*2*^ = 87%). We performed a subgroup analysis to see whether this heterogeneity was due to differences in patients’ lesion location. Results confirmed that learning ability differed as a function of lesion location (Chi^2^ = 20.66, *p* = .0001; *I*^2^ = 86%). Specifically, only patients with isolated subcortical lesions did not show significant learning (MD = 37.7 ms, [-69.0, 144.4], Z = 0.69, *p* = .49). Too few studies were available to further explore possible other causes of the statistical heterogeneity.

We descriptively synthesized the results of Orrell et al. [77] and Boyd and Winstein [50]. Of these two the results of Orrell et al. [77] seem largely in line with findings of the above meta-analysis. Specifically, they found that patients with supratentatorial brain damage demonstrated learning; at the end of two days of practice, patients’ reaction times were 96 ms faster for the repeated than for the random blocks. Although the exact significance of this learning effect is unclear, its magnitude seems in line with the findings of our meta-analysis of studies with mixed cortical/subcortical patient populations (Fig 3).

The results of Boyd and Winstein [50] seemed to deviate from those of the meta-analysis, though. In this study, patients with lesions in the supratentatorial subcortex practiced a CTT task on three consecutive days. Different from their peers in the meta-analysis, patients demonstrated significant learning, as evidenced by less tracking error on repeated versus random stimuli (ΔRMSE = 6.4%, SE = 0.98, *t*(1,4) = 6.5; *p* < .01). We therefore performed a sensitivity analysis to check whether exclusion of Boyd and Winstein [50] influenced our meta-analysis. We transformed their learning score into milliseconds (based on the SMDs [59]) and added them to the meta-analysis. The pooled estimate did not change (MD = 70 ms, 95% CI = [46.2, 93.5], Z = 5.78, *p* < .00001; I^2^ = 86%), neither did the subgroup-analysis (Chi^2^ = 20.53, *p* = .0001; *I*^2^ = 85%). Thus, learning remained non-significant for the subcortical group, even when Boyd and Winstein’s findings were added to the analysis (MD = 52.77 ms, [-40.0, 145.5], Z = 1.12, *p* = .26).

**Fig 4:**
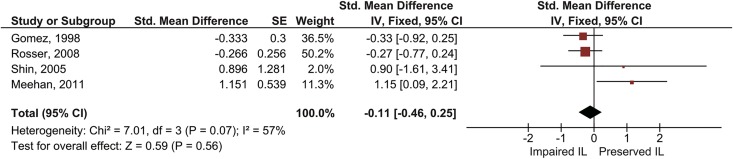
Pooled results of SRT-/CTT-studies that investigated implicit motor learning after stroke for the affected hand. Results concern the standardized mean differences in reaction time (in ms, for SRT-/SHM-studies) or RMSE (in percentages, for CTT-studies) between repeated and random blocks. Square size indicates a study’s relative contribution to the pooled estimate. Diamond width indicates the 95% confidence interval of the pooled effect. NB: CI = confidence interval; IL = implicit motor learning; IV = inverse variance; SE = standard error;

***3*.*4*.*1*.*2 SRT-Type tasks—Learning using the affected upper extremity***. We pooled the results of four studies that investigated implicit motor learning using the affected upper extremity, one of which used the CTT paradigm [79] and three the SRT-paradigm [51,54,70]. Two studies involved patients with isolated supratentatorial subcortical lesions [51,79], one study concerned a mixed patient population (mixed supratentatorial cortical/subcortical lesions [70]), and one study included isolated cerebellar lesions [54]. Data was pooled using a fixed effects model with the standardized mean difference in performance between repeated and random blocks as outcome measure. The pooled estimate showed no significant implicit motor learning (SMD = -.11, 95% CI [-.45, .25], Z = .59, *p* = .56; Fig 4). Not enough studies were available (N > 10) to analyze the moderate statistical heterogeneity (*I*^2^ = 57%, *p* = 0.07).

***3*.*4*.*1*.*3 SRT-Type tasks—Learning using both hands***. Four studies investigated implicit motor learning with SRT-paradigms that required bimanual responses [55,74–76]. The study of Vakil et al. [55] could not be pooled with the other three studies, as the variance of the learning effect could not be obtained. Its results are therefore presented separately and descriptively only.

**Fig 5:**
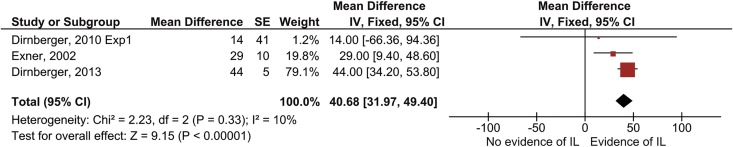
Pooled results of SRT-studies that investigated implicit motor learning after stroke with both hands. Results concern the mean differences in reaction time (in ms) between repeated and random blocks. Square size indicates a study’s relative contribution to the pooled estimate. Diamond width indicates the 95% confidence interval of the pooled effect. NB: CI = confidence interval; Exp1 = experiment 1; IL = implicit motor learning; IV = inverse variance; SE = standard error;

Of the three studies that were pooled two included patients with isolated cerebellar lesions [74,75], while one studied patients with isolated supratentatorial subcortical lesions [76]. We pooled results using a fixed effects model with the mean difference in reaction time between repeated and random blocks as outcome measure (Fig 5). Overall, learning was significant (MD = 40.7 ms, 95% CI [32.0, 49.4], Z = 9.2, *p* < .00001). Statistical heterogeneity was negligible (*I*^2^ = 10%).

In the study by Vakil et al. [55] patients with isolated supratentatorial subcortical lesions practiced the SRT-task within one day. At the end of practice, patients responded faster (36 ms) on repeated than on random blocks. Although it is unclear whether this finding was statistically significant, the magnitude of the effect seems similar to the meta-analysis of the other three studies.

***3*.*4*.*1*.*4 Motor tasks with more ‘complex’ movement dynamics***. As noted earlier, only one study investigated implicit motor learning abilities after stroke on a whole body task (Orrell et al. [32]). In this study five patients (a mix of patients with supra- and subtentatorial lesions) practiced a balance board task. Implicit motor learning was induced by means of an errorless learning approach, by progressively increasing task difficulty through reduction of the balance board’s rotational resistance across practice. Balance performance significantly improved after practice, an improvement that was maintained up to one week later at a delayed retention test (F(2,17) = 2.64, *p* = .10).

#### 3.4.2 Research question 2: Is patients’ implicit motor learning ability impaired compared to that of healthy peers?

***3*.*4*.*2*.*1 SRT-Type tasks—Learning using the unaffected upper-extremity***. Twelve studies contrasted implicit motor learning involving the unaffected hand in patients with healthy controls. Ten of these were eligible for data pooling. One study by Boyd and Winstein [50] apparently concerned the same control group as an earlier study of Boyd and Winstein [47]. As we could not include the same control group twice in our analysis, a computer randomly determined which results to include in the meta-analysis (i.e., in this case Boyd & Winstein [47]).

**Fig 6:**
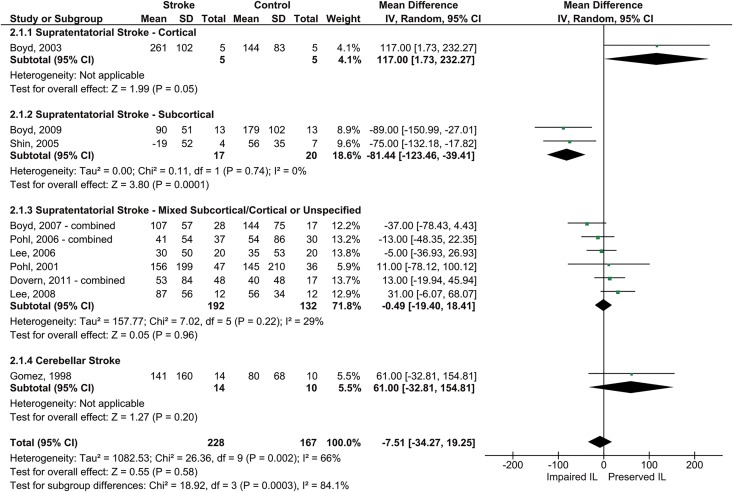
Pooled results of SRT-/SHM-studies that compared implicit motor learning for the unaffected hand between patients with stroke and healthy controls. Results concern the mean differences in reaction time (in ms) between repeated and random blocks for both groups. Square size indicates the study’s sample size. Diamond width indicates the 95% confidence interval of the pooled effect. Note that we collapsed the data for studies that included multiple stroke patient groups, as these studies only incorporated one healthy control group (following Cochrane recommendations [59]). NB: CI = confidence interval; IL = implicit motor learning; IV = inverse variance; SD = standard deviation;

For our meta-analysis, we pooled the 10 studies’ resultswith a random effects model. Themean difference in reaction time between random and repeated blocks served as outcome measure (Fig 6). Overall, patients demonstrated unimpaired implicit motor learning with their unaffected hand (MD = -7.5 ms, 95% CI = [-34.3, 19.2], Z = .55, *p* = .58). Considerable statistical heterogeneity was present (*I*^*2*^ = 66%). As for the first research question, subgroup analyses revealed that this may in part be due to the fact that learning ability differed as a function of lesion location (Chi^2^ = 18.9, *p* = .0003): Implicit motor learning was significantly impaired in patients with isolated supratentatorial subcortical lesions (MD = -81.4 ms, [-123.5, -39.4], Z = 3.8, *p* = .0001), but not in the other patient groups. Additional causes for the statistical heterogeneity could not be explored.

The results of Orrell et al. [77] differ slightly from those of the meta-analysis, as they found that patients with supratentatorial brain damage showed less pronounced learning than healthy controls (i.e., 96 ms for stroke vs.177 ms for controls at the end of day 2; F(2,12) = 6.93; *p* < .01).

**Fig 7:**
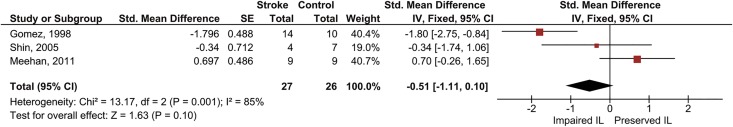
Pooled results of SRT-/CTT-studies that compared implicit motor learning for the affected hand between patients with stroke and healthy controls. Results concern the standardized mean differences in reaction time (in ms, for SRT-studies) or RMSE (in percentages, for CTT-studies) between repeated and random blocks. Square size indicates a study’s relative contribution to the pooled estimate. Diamond width indicates the 95% confidence interval of the pooled effect. NB: CI = confidence interval; IL = implicit motor learning; IV = inverse variance; SE = standard error;

***3*.*4*.*2*.*2 SRT-Type tasks—Learning using the affected upper-extremity*.** Three studies contrasted cerebellar [54] and supratentatorial subcortical [51,79] patients’ implicit motor learning abilities using the affected upper extremity with healthy controls. One study used the CTT paradigm [79] and 2 studies used the SRT-paradigm [51,54]. We pooled the results of these studies using a fixed effects model with standardized mean difference in performance between repeated and random blocks as outcome measure (Fig 7). Implicit motor learning of patients was not significantly different from controls (SMD = -.51, 95% CI [-1.1, .10], Z = 1.63, *p* = .10). Too few studies (N < 10) were available to investigate the considerable statistical heterogeneity (*I*^*2*^ = 85%).

***3*.*4*.*2*.*3 SRT-Type tasks—Learning using both hands***. Four studies compared patients’ implicit motor learning abilities with those of healthy controls, all of them using SRT-paradigms that require bimanual responses [55,74–76]. Similar to the first research question, the study of Vakil et al. [55] is discussed separately in a descriptive fashion.

**Fig 8:**
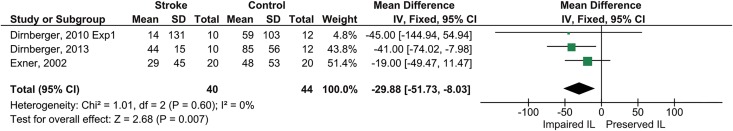
Pooled results of SRT-studies that compared implicit motor learning with both hands by patients with stroke and healthy controls. Results concern the mean differences in reaction time (in ms) between repeated and random blocks. Square size indicates a study’s sample size. Diamond width indicates the 95% confidence interval of the pooled effect. NB: CI = confidence interval; Exp1 = experiment 1; IL = implicit motor learning; IV = inverse variance; SD = standard deviation;

The three studies that were pooled either involved cerebellar [74,75] or supratentatorial subcortical [76] patients. We pooled results using a fixed effects model with the mean difference in reaction time between repeated and random blocks as outcome measure (Fig 8). Overall, implicit motor learning was found to be significantly impaired (MD = -29.9 ms, 95% CI [-51.7, -8.0], Z = 2.68, *p* = .007). No statistical heterogeneity was noted (*I*^2^ = 0%).

The results of Vakil et al. [55] confirm the results of the meta-analysis. Patients with lesions in the supratentatorial subcortex showed impaired learning (36 ms) compared to healthy controls (97 ms) as evidenced by a significant Group by Block interaction (F(1,30) = 5.96; *p* < .05).

***3*.*4*.*2*.*4 Motor tasks with more ‘complex’ movement dynamics***. Results of Orrell et al. [32] showed that patients who engaged in errorless learning during balance training showed similar improvement in and retention of balancing performance as did healthy controls (i.e., no significant interaction; F(2,17) = 0.39; *p* = .70).

#### 3.4.3 Research question 3: Is implicit motor learning more or less impaired than explicit motor learning following stroke?

**Fig 9:**
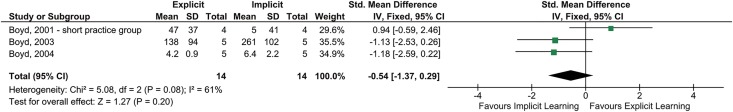
Pooled results for SRT-/CTT-studies that compared the effectiveness of implicit and explicit motor learning for the unaffected hand post-stroke. Results concern the standardized mean differences in reaction time (in ms, for SRT-studies) or RMSE (in percentages, for CTT-studies) between repeated and random blocks. Square size indicates a study’s relative contribution to the pooled estimate. Diamond width indicates the 95% confidence interval of the pooled effect. NB: CI = confidence interval; IV = inverse variance; SD = standard deviation;

***3*.*4*.*3*.*1 SRT-Type Tasks—Learning using the unaffected upper-extremity***. All identified SRT-type studies that contrasted implicit and explicit motor learning post-stroke concerned learning with the unaffected hand [47,50,52]. No studies were found that focused on learning using the more affected extremity. The three studies contrasted implicit and explicit motor learning abilities of patients with isolated subcortical [50], cortical [47], and mixed subcortical/cortical supratentatorial lesions [52] (Fig 9). Two studies used a SRT-paradigm [47,52] while one study used a CTT paradigm [50]. We pooled their results with a fixed effects model with the standardized mean difference in performance between random and repeated blocks as outcome measure. Overall, implicit learning did not result in superior learning compared to explicit learning (SMD = -.54, 95% CI[-1.37, .29], Z = 1.27, *p* = 0.20; Fig 9). Considerable heterogeneity was present (61%), but could not be further investigated.

***3*.*4*.*3*.*2 Motor tasks with more ‘complex’ movement dynamics***. In line with the above meta-analysis, the study by Orrell et al. [32] reported that patients who had implicitly learned the balancing task (with errorless learning) demonstrated a similar improvement in balance skill as those patients who had explicitly learned this task (through discovery learning). Specifically, at the delayed retention test one week post-practice, the implicit group’s performance did not significantly differ from that of the explicit group (M_Implicit_ = 8.8±1.5 RMSE; M_Explicit_ = 9.0±0.6 RMSE; *t*(1,8) = 0.28; *p* = .79).

## 4. Discussion

### 4.1 Principal findings

The aim of this review was to determine the extent to which implicit motor learning is possible after stroke. Specifically, we investigated whether patients with stroke could significantly improve their motor performance through implicit motor learning, as well as how patients’ implicit motor learning abilities compare to healthy peers. Furthermore, we scrutinized evidence to determine whether implicit motor learning is more or less effective than explicit motor learning post-stroke.

In total, we identified 20 studies that investigated implicit motor learning after stroke. Of note, limited information was available on implicit motor learning in clinically relevant settings. Specifically, only one study [32] investigated implicit motor learning in a clinically relevant balancing task, but all other studies concerned adaptations of the classic SRT-paradigm. Relatedly, the majority of studies investigated learning with the relatively unaffected side, and only few studies were concerned with patients’ ability to learn with their affected or paretic side (i.e., four studies [51,54,70,79] concerned the affected extremity, while five studies [32,55,74–76] required bilateral involvement).

The meta- and descriptive syntheses suggested that patients generally show significant and unimpaired implicit motor learning with their unaffected hand. An exception may be patients with subcortical lesions, as they overall did not demonstrate significant learning and were significantly impaired compared to healthy peers. Learning tended to be less consistent and more impaired when the paretic hand was involved. Based on the four studies that contrasted implicit motor learning with explicit motor learning, it may be that both modes of learning are equally preserved after stroke.

However, as of yet any conclusions regarding the above findings must be considered premature, due to three main reasons. First and foremost, reliable interpretation of these findings is compromised due to the overall high risk of bias that was noted across studies. This bias was mostly due to insufficient reporting on participant selection, explicit knowledge, and group comparability. Second, studies were generally of limited clinical relevance, since almost all concerned SRT-type tasks and/or only focused on patients’ ability to learn with their relatively unaffected side. Finally, most studies consisted of quite small sample sizes (i.e., *M* = 14 patients per group). As a result, it is yet premature to draw any conclusions regarding implicit motor learning abilities of people with stroke, let alone regarding its effectiveness and suitability for clinical practice. Nevertheless, the current findings seem appropriate as a starting point for building hypotheses for future research. Below, we will discuss these hypotheses as well as other implications of our findings for research and clinical practice. First, though, we will shortly discuss the reasons for the risk of bias among the included studies.

### 4.2 Risk of bias assessment

With the exception of two studies [47,50], all studies were subject to a moderate to high risk of bias. This was due to a variety of reasons. First, all but one study [70] failed to clearly describe the screening and selection of subjects, while most studies also lacked proper description of participants’ characteristics (e.g., in terms of motor and/or cognitive functioning)[51,52,55,68,69,72]. Further, a significant limitation of those studies that contrasted implicit motor learning of patients with healthy controls is the lack of information regarding group comparability. Confounders such as motor or cognitive functioning often were neither reported for the patient and control groups [47,49,50,55,68,69,71,73,75–79], nor matched across groups or corrected for in the analysis of implicit motor learning [47,49–51,68,69,71–73,77,79]. This makes it difficult to assess the representativeness of patients of the general stroke population and the comparability of stroke and control groups, resulting in a high risk of selection bias. We acknowledge that it may be challenging to find appropriate measurement scales to reliably compare stroke patients’ motor abilities with those of healthy elderly, as the latter will generally achieve maximum scores on stroke-specific instruments, such as the Fügl-Meyer Assessment or Motricity Index. To circumvent this problem, some studies in our review used initial performance during the first block of practice as a measure of baseline motor ability. However, this is not a valid method, as these values will be influenced by the experimental manipulation. Therefore, we strongly recommend to incorporate task-relevant alternatives that do not have a ceiling effect, such as the fast-tapping task used in the SRT-study by Shin et al [51]. Alternatively, authors may also conduct pre-test measurements of the to-be learned motor task. When groups differ in motor ability, a statistical correction for motor ability is warranted, for instance by means of analysis of covariance.

On a different note, the risk of so-called performance bias was high as well. For several studies we could not determine the likelihood that patients indeed learned implicitly rather than explicitly, either because only very superficial explicit knowledge checks were used–merely probing patients whether they noticed anything about the task [54,68–70]^–^or because it was unclear if patients’ explicit knowledge exceeded chance levels [49,51,72,78]. In other studies patients had acquired so much explicit task-related knowledge that it is not unlikely that they at least partially engaged in explicit motor learning [55,71,73,77].

A final limitation of a considerable number of studies was that practice sessions were of very short duration–i.e., learning was assessed within a single practice session within one day, without delayed retention tests [49,51,54,69–72,74–76]. This can be problematic for two reasons. First, such a short practice period might have limited these studies’ power to find significant learning effects, as implicit learning is considered to be a relatively slow process [27,80]. Second, learning effects that are observed immediately after practice can substantially differ from those assessed following a delay period (i.e., > 24 hours following the end of practice [81,82]). This latter issue may also have contributed to the statistical heterogeneity noted in our meta-analyses.

Overall, the points outlined above added up to a considerable risk of bias in most studies.

### 4.3 Implications for research

The findings of this review largely leave unanswered our previous question, but allow further specification of these questions for future studies to answer: (1) Do patients with stroke remain able to learn clinically relevant, complex motor tasks with their affected side in an implicit way?; (2) Are implicit and explicit motor learning equally preserved post-stroke?; and (3) How do different lesion locations (and especially subcortical lesions) affect the effectiveness of implicit and explicit motor learning post-stroke?

Considering the risk of bias issues outlined in the previous section, we recommend that studies that investigate these and other hypotheses regarding implicit motor learning post-stroke comprehensively report their procedures and findings, using checklists like the STROBE and CONSORT statements [83,84]. Studies should especially include proper manipulation checks, by documenting the amount of participants’ explicit movement-related knowledge after practice. Another seemingly obvious, yet currently often not met requirement for further studies is to incorporate appropriate sample sizes, preferably based on power analysis

On a different note, future studies should consider the clinical relevance of the to-be learned motor task. As highlighted by the current review, studies into implicit motor learning after stroke have mostly been restricted to SRT-type paradigms, in which patients practiced with their relatively unaffected hand over a relatively short period of time. The results obtained with these types of paradigms may not be easily generalizable to more complex motor tasks of daily living (i.e., walking, grasping, and balancing [85]). Therefore, for implicit motor learning to have any clinical utility it must be determined whether patients post-stroke are able to learn these more complex motor tasks in an implicit way. To this end, future studies should test the effectiveness and feasibility of the implicit learning paradigms briefly outlined in the introduction: dual-task learning [17], errorless learning [31], analogy learning [29], and external focus learning [33]. These paradigms have been shown to successfully effectuate implicit motor learning across a wide range of tasks in non-neurologically impaired individuals [27–35], but remain virtually untested in people with stroke. Further, for greater clinical relevance, outcome measures outside the context of the trained motor task should be incorporated (e.g., dual-task performance, fall-risk, patient reported outcome measures, quality of life questionnaires). Also, these implicit learning methods need to be contrasted with explicit motor learning—which seems the “default” mode of learning during physical therapy post-stroke [22,23]. Finally, researchers may also want to consider the stratification of patients according to their lesion location, to assess if and how lesion location influences the effectiveness of implicit (and explicit) motor learning interventions (especially focussing on the influence of subcortical damage).

The above recommendations can be best implemented in randomized controlled trials (RCTs) that compare the effectiveness of implicit and explicit motor learning interventions post-stroke.

### 4.4 Implications for practice

As of yet, it remains unclear to what extent implicit motor learning is possible in people with stroke. Also there is a significant lack of studies that investigated implicit motor learning on tasks of greater complexity in movement dynamics and with more clinical relevance than the SRT-paradigm. Therefore, from a scientific point of view, the implementation of implicit motor learning techniques in rehabilitation therapy post-stroke is premature. This is not to say that therapists should refrain from exploring interventions that promote implicit–or explicit–motor learning when treating their patients. Several of the abovementioned techniques (dual-task learning, errorless learning, analogy learning, learning with external focus instructions) may well prove useful, if only to expand a therapist’s toolbox in treating his/her patients. In this light the case-series by Kleynen et al. [86] may be of interest, as it illustrates how analogy learning can be used to improve gait in people with stroke. In any event, it is important that therapists are aware that the effectiveness of any of these interventions to promote implicit motor learning in people with stroke has not yet been proven.

### 4.5 Strengths and limitations

This study is the first to systematically review implicit motor learning in people with stroke. The sensitive search strategy allowed us to search as broad as possible, identifying papers from grey literature as well as from conventional databases. Also, rating the studies’ risk of bias aided the interpretation of the reliability and generalizability of the findings of this review. Nonetheless, several limitations should be noted. First, it cannot be ruled out that our review was subject to publication bias, in that we might have failed to identify non-significant and non-published studies. Also, as noted earlier, no full text could be obtained for two possibly relevant studies. It seems unlikely that this resulted in publication bias, though, since our funnel plot (S1 Fig) did not provide any indication of this. A second limitation of the current review concerns our inclusion criterion that studies needed to include a manipulation check as to the degree to which motor learning was more implicit or explicit. As a result, we excluded several clinically relevant studies that may potentially induce implicit motor learning. An example is augmented error-learning. It has been found that patients with asymmetric gait walk more symmetrically after a practice period in which they walked with even larger step length asymmetry, namely on a split belt treadmill with both sides set at different speeds [87,88]. Indeed, as long as patients are not consciously aware of these artificially enhanced errors, this intervention may trigger them to implicitly adapt their step length. However, the opposite may also be true, in that enforced errors may actually enhance patients’ awareness of their asymmetrical movement pattern, triggering them to explicitly correct it. The main point here is that without proper manipulation checks, we cannot tell which account holds true. Therefore, exclusion of studies that lacked these checks was warranted. A third limitation is the statistical heterogeneity that was present in most meta-analyses. Due to the limited number of studies we could often not further explore (i.e., by means of subgroup or meta-regression analysis) reasons for between-study variation in learning effect. In the two cases that exploration of heterogeneity was possible, we choose to group studies by lesion location, based on reports that some brain regions (like the subcortical basal ganglia [89,90]) may be more critical for implicit motor learning than others. Indeed, our decision to focus on this variable seems justified by the fact that lesion location indeed accounted for some of the statistical heterogeneity. However, our decision also meant that we were not in a position to further assess the possible role of other factors like studies’ risk of bias score, patients’ explicit knowledge, and duration of practice. A final limitation of this review concerns the risk of bias assessment. As of yet, there is no validated tool available to judge risk of bias in non-RCT’s. Nonetheless, the use of a modified Newcastle-Ottawa Scale (NOS) used in this review is considered to be the best alternative [59,91].

### 4.6 Conclusion

At this point, it remains unclear as to what degree implicit motor learning is possible after stroke. On a theoretical level, the application of implicit motor learning paradigms within rehabilitation practice post-stroke does still hold promise. Therefore, future research should focus on the effectiveness and feasibility of implicit motor learning in people with stroke, within clinically relevant contexts.

## Supporting Information

S1 Fig Funnel Plot of Included Studies. Studies were pooled for the main outcome (“Can patients with stroke learn motor tasks implicitly?”). For each study, its effect estimate (standard mean difference of performance in random versus repeated block; SMD) is plotted against its precision (standard error of the SMD; SE). The resulting symmetrical distribution of studies suggests that no publication bias was present.
(TIF)

S1 File Review Data. (RM5)

S1 Table Study Characteristics. (PDF)

S2 Table Prisma Checklist. (PDF)

S1 Text Search Strategy. Example of the search strategy for Medline.
(DOCX)

S2 Text **Appendices A-C with Modified Newcastle Ottawa Scales.** The three different Newcastle Ottawa Scales used to assess studies’ risk of bias for each of the three research questions. Of note, for each NOS-scale the items on performance bias rated studies’ quality on their success of blinding participants (i.e., the amount of explicit knowledge that participants gained with practice). In the NOS-scales used in this study, these items were given extra weight (i.e., 2 points could be scored per item, instead of 1), as it is the hallmark of implicit motor learning that learners do not gain explicit movement-related knowledge. Studies in which learners gained considerable explicit knowledge run the risk of having measured a more explicit form of motor learning.
(DOCX)
