# Supplementary figures and images for: Is Implicit Motor Learning Preserved after Stroke? A Systematic Review with Meta-Analysis
Source: PLoS One. 2016 Dec 16;11(12):e0166376. doi: 10.1371/journal.pone.0166376 (PMC5161313; doi:10.1371/journal.pone.0166376)

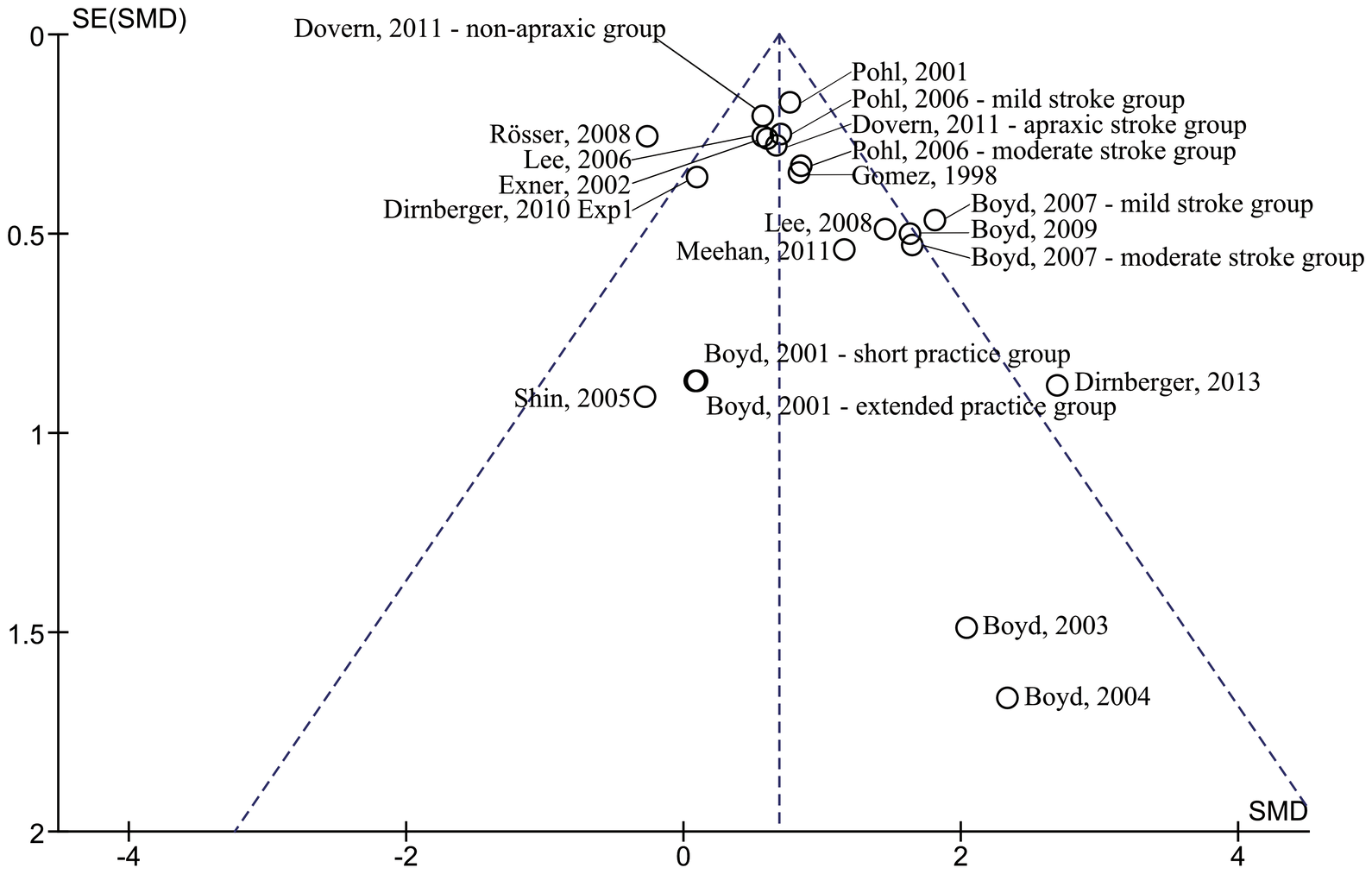

Supplement: S1 Fig — Studies were pooled for the main outcome (“Can patients with stroke learn motor tasks implicitly?”). For each study, its effect estimate (standard mean difference of performance in random versus repeated block; SMD) is plotted against its precision (standard error of the SMD; SE). The resulting symmetrical distribution of studies suggests that no publication bias was present. (TIF) [file pone.0166376.s001.tif]
