# Supplementary material for: Is Implicit Motor Learning Preserved after Stroke? A Systematic Review with Meta-Analysis
Source: PLoS One. 2016 Dec 16;11(12):e0166376. doi: 10.1371/journal.pone.0166376 (PMC5161313; doi:10.1371/journal.pone.0166376)
Supplement: S1 Table — (PDF) [file pone.0166376.s003.pdf]

S1 Table. Study Characteristics.

Author / Experimental groups	Inclusion criteria	Group characteristics	Motor task	Outcome measure	Declarative knowledge
Boyd & Winstein [52]<br><br><br>- Stroke group (Implicit learning – short practice)<br><br>- Stroke group (Implicit learning- extended practice)<br><br>- Stroke group (Explicit learning– short practice)	Stroke:<br>- ≥ 6 months since stroke<br>- Unilateral damage to sensorimotor brain areas	Stroke (implicit – short practice):<br>- *N* (m/f) = 4 (3/1)<br>- Age (y) = 54 ± 16<br>- Months since stroke = 60 ± 45<br>- Stroke location = (sub)cortical-SupT<br>- Lesion side (L/R) = (3/1)<br>- MMSE = 28.0 ± 0.8<br>- Motor functioning = ?<br><br>Stroke (implicit - extended practice)<br>- *N* (m/f) = 4 (4/0)<br>- Age (y) = 59 ± 14<br>- Months since stroke = 7 ± 1<br>- Stroke location = (sub)cortical-SupT<br>- Lesion side (L/R) = (3/1)<br>- MMSE = 28.3 ± 1.1<br>- Motor functioning = ?<br><br>Stroke (explicit – short practice)<br>- *N* (m/f) = 4 (2/2)<br>- Age (y) = 55 ± 4<br>- Months since stroke = 13 ± 7<br>- Stroke location = (sub)cortical-SupT (3) & Pons (1)<br>- Lesion side (L/R) = SMC(3/0)/Pons(0/1)<br>- MMSE = 27.8 ± 1.5<br>- Motor functioning = ?	SRT task<br><br>Procedure:<br>Block = 6 repetitions of 9-item sequence<br><br>**Day 1**<br>6 blocks:<br>- Blocks 1 & 5: random<br>- Blocks 2-4 & 6: repeated<br><br>For the extended practice group, the above procedure was repeated on day 2 and day 3<br><br>Hand used:<br>Ipsilesional hand	Average median response time (ms) per block<br><br>Implicit motor learning:<br>Mean difference in reaction time between block 5 (random) and 6 (repeated) at the last day of practice	**Tests used:**<br>- Awareness (% of participants)<br>- Recognition (% correct)<br>- Recall (% correct)<br>- Chance = 25%)<br><br>**Results:**<br>Stroke (implicit – short practice):<br>Aware = 25%<br>Recognition = 0%<br>Recall = 8% ± 14<br><br>Stroke (implicit - extended practice):<br>Aware = 0%<br>Recognition = 0%<br>Recall = 17% ± 17<br><br>Stroke (explicit – short practice):<br>Recognition = 100%<br>Recall = 50% ± 29
Boyd & Winstein [44]	Stroke:	Stroke (Implicit):	SRT task	Average median response time	**Tests used:**

- Stroke group (Implicit learning)<br><br>- Stroke group (Explicit learning)<br><br>- Control group (Implicit learning)<br><br>- Control group (Explicit learning)	- ≥ 6 months since stroke<br>- Unilateral damage to sensorimotor cortex (MCA)<br>- Right hand dominant<br>- MMSE > 25<br>- No acute medical problems<br>- No uncorrected visual impairment<br>- No history of psychiatric admission, or neurologic impairment<br><br><u>Control:</u><br>- Same criteria + no neurological impairment	- *N* (m/f) = 5 (4/1)<br>- Age (y) = 59 ± 19<br>- Months since stroke = 48 ± 30<br>- Stroke location = Cortical-MCA<br>- Lesion side (L/R) = 4/1<br>- MMSE = 27.8 ± 1.8<br>- FMA-UE (0-66) = 27 ± 19<br><u>Stroke (Explicit):</u><br>- *N* (m/f) = 5 (2/3)<br>- Age (y) = 59 ± 11<br>- Months since stroke = 33 ± 19<br>- Stroke location = Cortical-MCA<br>- Lesion side (L/R) = 2/3<br>- MMSE = 29.0 ± 1.2<br>- FMA-UE (0-66) = 30 ± 21<br><br><u>Control (Implicit):</u><br>- *N* (m/f) = 5 (2/3)<br>- Age (y) = 57 ± 16<br>- MMSE = 29.6 ± 0.5<br><u>Control (Explicit):</u><br>- *N* (m/f) = 5 (1/4)<br>- Age (y) = 55 ± 11<br>- MMSE = 29.8 ± 0.4	<u>Procedure:</u><br>Block = 10 repetitions of 10-item sequence<br><br>**Days 1-3:**<br>7 blocks:<br>- Blocks 1 & 6: random<br>- Blocks 2-5 & 7: repeated<br>**Day 4:**<br>- 1 repeated & 1 random block<br><br><u>Hand used:</u><br>Stroke: ipsilesional hand<br>Control: matched to stroke groups	(ms) per block<br><br><u>Implicit motor learning:</u><br>Mean difference in response time between the repeated block at retention and the random block at end of day 1	- Awareness (% of participants)<br>- Recognition (% correct)<br>- Chance = 50%<br>- Recall (% correct)<br>- Chance = 50%<br><br>**Results:**<br><u>Stroke (Implicit):</u><br>Aware = 20%<br>Recognition = 53%<br>Recall = 33%<br><br><u>Stroke (Explicit):</u><br>Recognition = 73%<br>Recall = 53%<br><br><u>Control (Implicit):</u><br>Aware = 80%<br>Recognition = 66%<br>Recall = 40%<br><br><u>Control (Explicit):</u><br>Recognition = 100%<br>Recall = 86%
Boyd & Winstein [50]<br><br>- Stroke group (Implicit learning)<br><br>- Stroke group (Explicit learning)<br><br>- Control group (Implicit learning)<br><br>- Control group (Explicit learning)	<u>Stroke:</u><br>- ≥ 6 months since stroke<br>- Unilateral BG damage<br>- Right hand dominant<br>- MMSE > 25<br>- No acute medical problems<br>- No uncorrected visual impairment<br>- No history of psychiatric admission, or neurologic impairment<br><br><u>Control:</u><br>- Same criteria + no neurological impairment	<u>Stroke (Implicit):</u><br>- *N* (m/f) = 5 (3/2)<br>- Age (y) = 58 ± 15<br>- Months since stroke = 10 ± 6<br>- Stroke location = Subcortical-BG<br>- Lesion side (L/R) = 1/4<br>- MMSE = 28.4 ± 1.1<br>- FMA-UE (0-66) = 44 ± 16<br><u>Stroke (Explicit):</u><br>- *N* (m/f) = 5 (4/1)<br>- Age (y) = 51 ± 10<br>- Months since stroke = 28 ± 28	CT task<br><br><u>Procedure:</u><br>Block = 10 trials of tracking (30 seconds)<br><br>**Days 1-3:**<br>- 5 blocks of tracking<br>**Day 4:**<br>- Retention test: 1 block of tracking<br><br><u>Hand used:</u><br>Stroke: ipsilesional hand<br>Control: matched to stroke	Average root-mean-squared error (RMSE) of tracking for random and repeated segments per block<br><br><u>Implicit motor learning:</u><br>Mean difference in RMSE during tracking of repeated segments at retention and tracking of random segments at the end of day 1	**Tests used:**<br>- Awareness (% of participants)<br>- Recognition (% correct)<br>- Chance = 50%<br>- Recall (% correct)<br>- Chance = 33%<br><br>**Results:**<br><u>Stroke (Implicit):</u><br>Aware = 0%<br>Recognition = 46% ± 15<br>Recall = 0%<br><br><u>Stroke (Explicit):</u><br>Recognition = 40% ± 28

		- Stroke location = Subcortical-BG<br>- Lesion side (L/R) = 1/4<br>- MMSE = 28 ± 1.4<br>- FMA-UE (0-66) = 48 ± 20<br><br><u>Control (Implicit):</u><br>- *N* (m/f) = 5 (2/3)<br>- Age (y) = 57 ± 16<br>- MMSE = 29.6 ± 0.5<br><u>Control (Explicit):</u><br>- *N* (m/f) = 5 (1/4)<br>- Age (y) = 55 ± 11<br>- MMSE = 29.8 ± 0.4	groups		<u>Control (Implicit):</u><br>Aware = 0%<br>Recognition = 66% ± 33<br>Recall = 20%<br><br><u>Control (Explicit):</u><br>Recognition = 73% ± 33
Boyd et al. [71]<br><br>- Mild stroke group<br>- Moderate stroke group<br>- Control group	<u>Stroke:</u><br>- ≥ 6 months since stroke<br>- Unilateral brain lesion<br>- Right hand dominant<br>- MMSE > 25<br>- No acute medical problems<br>- No UE pathology<br>- No uncorrected visual impairment<br>- No history of psychiatric admission, or neurologic impairment<br><br><u>Control:</u><br>- No neurological impairment<br>- Age-matched	<u>Stroke (mild):</u><br>- *N* (m/f) = 16 (10/6)<br>- Age (y) = 54 ± 4<br>- Months since stroke = ?<br>- Stroke location = (sub)cortical-SupT<br>- Lesion side (L/R) = 9/7<br>- MMSE = 29.5 ± 0.8<br>- Motor functioning = ?<br>- Orpington score = 2.3 ± 0.1<br><u>Stroke (moderate):</u><br>- *N* (m/f) = 12 (5/7)<br>- Age (y) = 61 ± 3<br>- Months since stroke = ?<br>- Stroke location = (sub)cortical-SupT<br>- Lesion side (L/R) = 5/7<br>- MMSE = 28.8 ± 1.2<br>- Motor functioning = ?<br>- Orpington score =3.4 ± 0.2<br><br><u>Control:</u><br>- *N* (m/f) = 17 (6/11)<br>- Age (y) = 53 ± 3<br>- MMSE = 29.7 ± 0.7	SHM task<br>SRT task<br><br><u>Procedure:</u><br>- SHM & SRT:<br>Block = 10 repetitions of 10-item sequence<br><br>**Day 1:**<br>Both tasks: 12 blocks<br>- Block 1 & 11: random<br>- Blocks 2-10 & 12: repeated<br><br><u>Hand used:</u><br>Stroke: ipsilesional hand<br>Control: matched to stroke groups	Both tasks:<br>Average median response time (ms) per block<br><br><u>Implicit motor learning:</u><br>Both tasks: Mean difference in response time between (repeated) block 12 and (random) block 11	**Tests used:**<br>- Awareness (% of participants)<br>- Recognition (% correct)<br>- Chance = 50%<br>- Recall (% correct)<br>- Chance = 25%<br><br>**Results:**<br><u>Stroke (Mild):</u><br>SHM: Aware = 81%<br>SHM: Recognition = 64% ± 30<br>SHM: Recall = 57% ± 14<br>SRT: Aware = 56%<br>SRT: Recognition = 64% ± 12<br>SRT: Recall = 51% ± 19<br><br><u>Stroke (Moderate):</u><br>SHM: Aware = 85%<br>SHM: Recognition = 72% ± 15<br>SHM: Recall = 44% ± 22<br>SRT: Aware = 62%<br>SRT: Recognition = 56% ± 21<br>SRT: Recall = 37% ± 15<br><br><u>Control:</u><br>SHM: Aware = 82%<br>SHM: Recognition = 81% ± 16<br>SHM: Recall = 69% ± 17

					SRT: Aware = 71%<br>SRT: Recognition = 66% ± 19<br>SRT: Recall = 59% ± 22
Boyd et al. [73]<br><br>- Stroke group<br>- Control group	<u>Stroke:</u><br>- ≥ 6 months since stroke<br>- Damage to BG<br>- MMSE > 25th percentile<br>- No uncorrected visual impairment<br>- No orthopedic condition interfering with task performance<br><br><u>Control:</u><br>- Same criteria + no neurological impairment	<u>Stroke:</u><br>- *N* (m/f) = 13 (8/5)<br>- Age (y) = 59 ± 16<br>- Months since stroke = 60 ± 53<br>- Stroke location = Subcortical-BG<br>- Lesion side (L/R) = 2/11<br>- MMSE = 28.3 ± 2<br>- FMA-UE (0-66) = 34 ± 18<br>- Orpington score = 2.8 ± 0.7<br><br><u>Control:</u><br>- *N* (m/f) = 13 (5/8)<br>- Age (y) = 60 ± 16<br>- MMSE = 29.8 ± 0.6	SRT task<br><br><u>Procedure:</u><br>Block = 10 repetitions of 12-item sequence<br><br>**Days 1&2**<br>6 blocks:<br>- Block 1: random<br>- Blocks 2-6: repeated<br>**Day 3:**<br>- Retention test: 1 repeated block<br><br><u>Hand used:</u><br>Stroke: ipsilesional hand<br>Control: matched to stroke group	Average median response time (ms) per block<br><br><u>Implicit motor learning:</u><br>Mean difference in response time between repeated block at retention, and last random block on day 2	**Tests used:**<br>- Awareness (% of participants)<br>- Recognition (% correct)<br>- Chance = 50%<br>- Recall (% correct)<br>- Chance = 25%<br><br>**Results:**<br><u>Stroke:</u><br>Aware = 85%<br>Recognition-true = 67% ± 41<br>Recognition-false = 68% ± 30<br>Recall = 52% ± 20<br><br><u>Control:</u><br>Aware = 77%<br>Recognition-true = 82% ± 22<br>Recognition-false = 83% ± 21<br>Recall = 52% ± 16
Dirnberger et al. (Exp. 1) [74]<br><br>- Stroke group<br>- Control group	<u>Stroke:</u><br>- > 6 months since stroke<br>- Isolated cerebellar lesion<br>- No other cerebral pathology<br>- No history of neurological, psychiatric, or other relevant disease (e.g., arthritis)<br><br><u>Control:</u><br>- No neurological impairment	<u>Stroke:</u><br>- *N* (m/f) = 11 (5/6)<br>- Age (y) = 46 ± 15<br>- Months since stroke = 31 ± 18<br>- Stroke location = CB<br>- Lesion side (L/R/Bilateral) = 3/4/4<br>- MMSE = 29 ± 1<br>- ICARS = 6 ± 4<br>- PP (UL/UR/BL/BR) = 13±2/12±3/9±2/10±2<br><br><u>Control:</u><br>- *N* (m/f) = 13 (6/7)<br>- Age (y) = 45 ± 14<br>- MMSE = 29 ± 1<br>- PP (UL/UR/BL/BR) = 14±2/15±2/11±2/12±2	SRT task<br><br><u>Procedure:</u><br>Block = 9 repetitions of 10-item sequences<br><br>**Day 1:**<br>3 runs of 14 blocks:<br>- Blocks 1&2: random<br>- Blocks 3-7: repeated<br>- Blocks 8&9: random<br>- Blocks 10-14: interference<br>Followed by:<br>- 2 random & 5 repeated blocks<br><br><u>Hand used:</u><br>Stroke & Control: Middle and index finger of each	Average median response time (ms) per block<br><br><u>Implicit motor learning:</u><br>Mean difference in response time between final 5th repeated block and last preceding random block[a] at retention	**Tests used:**<br>- Awareness (% of participants)<br>- Recognition (% correct)<br>- Chance = 33%<br>- Recall (# items)<br><br>**Results:**<br><u>Stroke:</u> Awareness = 46%<br><u>Control:</u> Awareness = 62%<br><br><u>Both groups - Recognition & recall:</u><br>"No participant could recall the sequence, and both groups performed at chance when asked to identify the sequence out of three alternatives" (p. 1205)

			hand		
Dirnberger et al. [75]<br><br>- Stroke group<br>- Control group	Stroke:<br>- > 6 months since stroke<br>- Isolated cerebellar lesion<br>- No other cerebral pathology<br>- No history of neurological or psychiatric disease<br><br>Control:<br>- No neurological impairment	Stroke:<br>- *N* (m/f) = 10 (5/5)<br>- Age (y) = 47 ± 15<br>- Months since stroke = 28 ± 16<br>- Stroke location = CB<br>- Lesion side (L/R/Bilateral) = 3/4/3<br>- MMSE = 29 ± 1<br>- Motor functioning = ?<br><br>Control:<br>- *N* (m/f) = 13 (7/5)<br>- Age (y) = 43 ± 14<br>- MMSE = 29 ± 1<br>- Motor functioning = ?	SRT task<br><br>Procedure:<br>- Repeated/Test block = 45 repetitions of 10-item sequence<br>- Random block = 9 repetitions of random 10-item sequence<br><br>**Day 1:**<br>4 runs of 4 blocks:<br>- Block 1: random<br>- Block 2: repeated<br>- Block 3: random<br>- Block 4: test<br><br>Hand used:<br>Stroke & Control: Middle and index finger of each hand	Average median response time (ms) per block<br><br>Implicit motor learning:<br>Mean difference in response time between final repeated block (in run 4) and subsequent random block	**Tests used:**<br>- Awareness (% of participants)<br>- Recognition (% correct)<br>- Chance = 33%<br>- Recall (# items)<br><br>**Results:**<br>Stroke:<br>Aware = 50%<br>Recognition = 50%<br>Control:<br>Aware = 58%<br>Recognition = 33%<br><br>Both groups – Recall: "No participant could recall the sequence …" (p. 2212)
Dovern et al. [49]<br><br>- Apraxic stroke group<br><br>- Non-apraxic stroke group<br><br>- Control group	Stroke:<br>- First-ever left MCA-stroke<br>- > 8 days since stroke<br>- Right hand dominant<br>- For apraxic patients: Impaired in: imitating meaningless hand/finger positions, or imitating/actual object-use<br><br>Control:<br>- Healthy<br>- Age-matched	Stroke (apraxic):<br>- *N* (m/f) = 18 (9/9)<br>- Age (y) = 57 ± 12<br>- Days since stroke = 367 [16-1209]<br>- Stroke location =(sub)cortical-MCA<br>- Lesion side (L/R) = 18/0<br>- WM (CBTT) = 4.8<br>- ARAT (0-57) = 30<br>Stroke (non-apraxic):<br>- *N* (m/f) = 30 (22/8)<br>- Age (y) = 50 ± 12<br>- Days since stroke = 315 [27-1506]<br>- Stroke location =(sub)cortical-MCA<br>- Lesion side (L/R) = 30/0<br>- WM (CBTT) = 5.3	SRT task<br><br>Procedure:<br>Block = 10 repetitions of 6-item sequence<br><br>**Day 1:**<br>5 blocks:<br>- Blocks 1-4: repeated<br>- Block 5: different sequence with equal stimulus(-transition) probabilities as practiced sequence<br><br>Hand used:<br>Stroke & Control: left hand (ipsilesional/non-dominant hand)	Average median response time (ms) per block<br><br>Implicit motor learning:<br>Mean difference in response time between block 4 (repeated) and block 5 (random/unpracticed)	**Tests used:**<br>- Awareness (% of participants)<br>- Recall (# items)<br><br>**Results:**<br>Stroke (apraxic):<br>Recall = 2.7 ± 2.1 items<br><br>Stroke (non-apraxic):<br>Recall = 3.4 ± 2.0 items<br><br>Control:<br>Recall = 4.4 ± 1.3 items

		- ARAT (0-57) = 42<br><br>Control:<br>- *N* (m/f) = 17 (8/9)<br>- Age (y) = 54 ± 10<br>- WM (CBTT) = 5.4			
Exner et al. [76]<br><br>- Stroke group<br>- Control group	Stroke:<br>- Isolated BG-lesions<br>- ≥ 6 months since stroke<br>- < 70 years<br>- No history of psychiatric or neurological impairment<br><br>Control:<br>- No neurological impairment<br>- Matched for age, sex, & years of education	Stroke:<br>- *N* (m/f) = 20 (17/3)<br>- Age (y) = 53 ± 11<br>- Months since stroke = 24 ± 1.5<br>- Stroke location = Subcortical-BG<br>- Lesion side (L/R/Bilateral) = 9/9/2<br>- WAIS-R (IQ) = 100 ± 18<br>- General incoordination (no/mild/moderate) = 14/3/3<br><br>Control:<br>- *N* (m/f) = 20 (15/5)<br>- Age (y) = 52 ± 9<br>- WAIS-R (IQ) = 111 ± 18<br>- Motor functioning = ?	SRT task<br><br>Procedure:<br>Block = 10 repetitions of 12-item sequence<br><br>**Day 1:**<br>8 blocks:<br>- Blocks 1 & 6: random<br>- Block 2-5 & 7-8: repeated<br><br>Hand used:<br>Stroke & Control: middle and index finger of both hands	Average response time (ms) per block<br><br>Implicit motor learning:<br>Mean difference in response time between block 5 (repeated) and block 6 (random)	**Tests used:**<br>- Recall (# items)<br><br>**Results:**<br>Both groups:<br>“None of the groups scored significantly above random level” (p. 379)
Gómez-Beldarrain et al. [54]<br><br>- Stroke group<br>- Control group	Stroke:<br>- Isolated CB-lesions<br>- ≥ 6 months since stroke<br>- Right hand dominant<br>- No history of cognitive or neurological impairment<br><br>Control:<br>- No neuro(psycho)logical or physical impairment<br>- Not using any medication	Stroke:<br>- *N* (m/f) = 14 (10/4)<br>- Age (y) = 61 ± 11<br>- Months since stroke = 29 ± 22<br>- Stroke location = CB<br>- Lesion side (L/R) = 9/5<br>- WAIS-R (IQ) = N/A<br>- PP (UL/UR) = 11/12<br><br>Control:<br>- *N* (m/f) = 10 (7/3)<br>- Age (y)[range] = 62.6 [52-72]<br>- WAIS-R (IQ) = N/A<br>- PP (UL/UR) = 12/13.9	SRT task<br><br>Procedure:<br>Block = 10 repetitions of 10-item sequence<br><br>**Day 1:**<br>5 blocks:<br>- Blocks 1&5: random<br>- Block 2-4: repeated<br><br>Hand used:<br>Stroke & Control: Both hands tested separately	Median response time (ms) per block<br><br>Implicit motor learning:<br>Mean difference in response time between block 4 (repeated) and block 5 (random)	**Tests used:**<br>- Awareness (% of participants)<br><br>**Results:**<br>Stroke: Aware = 0%<br><br>Control: Aware = 20%<br><br>“None of the patients achieved explicit knowledge of the sequence and only two controls mentioned having noticed some sort of sequence, but were unable to reproduce the numbers” (p. 28)
Lee et al. [68]	Stroke:	Stroke:	SRT task	Average response time (ms)	**Tests used:**

 - Stroke group<br>- Control group	- Unilateral brain damage<br>- Korean MMSE > 24<br>- No hemianopsia/unilateral spatial neglect<br>- Right hand dominant<br><br>Control:<br>- No neurological impairment	- *N* (m/f) = 20 (12/8)<br>- Age (y) = 58 ± 12<br>- Months since stroke = 3.9 ± 2.6<br>- Stroke location = (sub)cortical-SupT<br>- Lesion side (L/R) = 8/12<br>- Korean MMSE = 27.1 ± 1.9<br>- Motor functioning = ?<br><br>Control:<br>- *N* (m/f) = 20 (11/9)<br>- Age (y) = 57 ± 7<br>- Cognitive/motor functioning = ?	 Procedure:<br>Block = 10 repetitions of 10-item sequence<br><br>**Day 1**<br>7 blocks:<br>- Block 1&6: random<br>- Block 2-5 & 7: repeated<br>**Day 2**<br>3 retention blocks:<br>- Blocks 1&3: random<br>- Block 2: repeated<br><br>Hand used:<br>Stroke: ipsilesional hand<br>Control: matched to stroke group	per block<br><br>Implicit motor learning:<br>Mean difference in response time between block 2 (repeated) and block 3 (random) at retention	- Awareness (% of participants)<br><br>**Results:**<br>Stroke: Aware = 35%<br>Control: Aware = 60%<br><br>Authors state that no subject could recall the exact order of stimuli (p.30-31)
Lee et al. [69]<br><br>- Stroke group<br>- Control group	Stroke:<br>- Unilateral brain damage<br>- < 3 months after stroke<br>- Korean MMSE > 24<br>- No hemianopsia, or unilateral spatial neglect<br>- Right hand dominant<br><br>Control:<br>- No neurological impairment	Stroke:<br>- *N* (m/f) = 12 (7/5)<br>- Age (y) = 62 ± 12<br>- Months since stroke = 1.9 ± 0.2<br>- Stroke location = (sub)cortical-SupT<br>- Lesion side (L/R) = 5/7<br>- Korean MMSE = 26.7 ± 0.4<br>- Motor functioning = ?<br><br>Control:<br>- *N* (m/f) = 12 (7/5)<br>- Age (y) = 57 ± 7<br>- Cognitive/motor functioning = ?	SRT task<br><br>Procedure:<br>Block = 10 repetitions of 12-item sequence<br><br>**Day 1**<br>7 blocks:<br>- Blocks 1&6: random<br>- Block 2-5 & 7: repeated<br><br>Hand used:<br>Stroke: ipsilesional hand<br>Control: matched to stroke group	Average response time (ms) per block<br><br>Implicit motor learning:<br>Mean difference in response time between block 5 (repeated) and block 6 (random)	**Tests used:**<br>- Awareness (% of participants)<br><br>**Results:**<br>Stroke: Aware = 33%<br>Control: Aware = 67%<br><br>[Authors state that no subject could recall the exact order of stimuli (p. 4)]
Meehan et al. [79]<br><br>- Stroke group<br>- Control group	Stroke:<br>- ≥ 12 months since stroke<br>- Subcortical stroke<br>- Right hand dominant<br>- MMSE > 25th percentile<br>- No earlier stroke<br>- No psychiatric, neurologic,	Stroke:<br>- *N* (m/f) = 9 (6/3)<br>- Age (y) = 64 ± 6<br>- Months since stroke = 53 ± 47<br>- Stroke location = Subcortical-SupT	CT task<br><br>Procedure:<br>Block = 10 trials of tracking (20 s)<br><br>**Day 1**:	Average root-mean-squared error (RMSE) of tracking for random and repeated segments for each block<br><br>Implicit motor learning:<br>Mean difference in RMSE for	**Tests used:**<br>- Recognition (%)<br>- Chance = 50%<br><br>**Results:**<br>Stroke: Recognition = 54%<br>Control: Recognition = 53%

	orthopedic or uncorrected visual impairment<br><br><u>Control:</u><br>- No neurological impairment<br>- Age- and sex-matched	- Lesion side (L/R) = 0/9<br>- MMSE = 29.3 ± 0.7<br>- FMA-UE (0-66) = 54 ± 12<br><br><u>Control:</u><br>- *N* (m/f) = 9 (4/5)<br>- Age (y) = 63 ± 7<br>- MMSE = 29.7 ± 0.5<br>- Motor functioning = ?	- 1 random & 1 repeated block<br>**Days 2-6:** 5 blocks<br>**Day 7:** Same as day 1<br><br><u>Hand used:</u><br>Stroke: contralesional hand<br>Control: left (non-dominant) hand	repeated and random segments at retention (day 7)	
Orrell et al. [32]<br><br>- Stroke group (Implicit/errorless learning)<br><br>- Stroke group (Explicit/discovery learning)<br><br>- Control group (Implicit/errorless learning)<br><br>- Control group (Explicit/discovery learning)	<u>Stroke:</u><br>- ≥ 12 months since stroke<br>- First-ever stroke<br>- MMSE > 24<br>- Discharged from all rehabilitation services<br><br><u>Control:</u><br>- No neurological impairment	<u>Stroke (errorless):</u><br>- *N* (m/f) = 5 (4/1)<br>- Age (y) = 49 ± 16<br>- Months since stroke = ?<br>- Stroke location & side = (sub)cortical-SupT = 4(2L/2R), CB=1<br>- MMSE = 26.8 ± 0.8<br>- BBS (0-56) = 38 ± 5.8<br><u>Stroke (discovery):</u><br>- *N* (m/f) = 5 (5/0)<br>- Age (y) = 55 ± 12<br>- Months since stroke = ?<br>- Stroke location = (sub)cortical-SupT<br>- Lesion side (L/R/Bilateral) = 1/3/1<br>- MMSE = 25.8 ± 1.3<br>- BBS (0-56) = 38 ± 9<br><br><u>Control (errorless):</u><br>- *N* (m/f) = 6 (3/3)<br>- Age (y) = 67 ± 9<br>- MMSE = 29.2 ± 0.7<br>- BBS (0-56) = 52 ± 1<br><u>Control (discovery):</u><br>- *N* (m/f) = 6 (3/3)<br>- Age (y) = 63 ± 5<br>- MMSE = 29.3 ± 0.78<br>- BBS (0-56) = 54 ± 1	Balance task:<br><br><u>Errorless learning:</u><br>Task difficulty progressively increased throughout practice<br><u>Discovery learning:</u><br>Task difficulty similar across trials; Participants need to discover verbal rules of how to perform task<br><br><u>Procedure:</u><br>Block = 1 trial of 60 seconds of balancing on balance board<br><br>**Day 1** (acquisition + post-test)<br>- 24 repeated blocks<br>- 4 blocks: ST-performance<br>- 4 blocks: DT-performance (kettle lift/number recall)<br><br>**Day 8** (delayed retention):<br>- 2 blocks ST-performance	Deviation from horizontal axis, expressed as average root-mean-squared error (RMSE)<br><br><u>Implicit motor learning:</u><br>Balance performance at delayed retention test	**Tests used:**<br>- # verbal movement-related rules<br><br>**Results:**<br><u>Stroke (errorless):</u><br>Number of rules: 1.4 ± 1.1<br><br><u>Stroke (discovery):</u><br>Number of rules: 3.4 ± 1.3<br><br><u>Control (errorless):</u><br>Number of rules: 1.8 ± 0.8<br><br><u>Control (discovery):</u><br>Number of rules: 2.7 ± 1.0
Orrell al. [77]<br><br>- Stroke group<br>- Control group	<u>Stroke:</u><br>- ≥ 12 months since stroke<br>- Hemiparesis<br>- Able to understand	<u>Stroke:</u><br>- *N* (m/f) = 7 (2/5)<br>- Age (y) = 60 ± 10<br>- Months since stroke = 35 ±	SRT task<br><br><u>Procedure:</u><br>Block = 10 repetitions of	Median response time (ms) per block<br><br><u>Implicit motor learning:</u>	**Tests used:**<br>- Awareness (% of participants)<br>- Recall/Prediction (# errors)<br>- Chance = 90 errors/30 correct

	instructions<br>- MMSE > 24<br>- Discharged from all rehabilitation services<br>- No hemianopsia or orthopedic impairment<br><br><u>Control:</u><br>- No neurological impairment	13<br>- Stroke location = (sub)cortical-AC<br>- Lesion side (L/R) = 0/7<br>- MMSE = 26.3 ± 1.0<br>- General motor impairment level (0-14) = 5.3 ± 2.4<br><br><u>Control:</u><br>- *N* (m/f) = 9 (4/5)<br>- Age (y) = 47 ± 9<br>- MMSE = 29.1 ± 1.1<br>- Motor functioning = ?	12-item sequence<br><br>**Days 1&2** (acquisition):<br>- Blocks 1-17 & 19-20: repeated<br>- Block 18: random<br>**Day 2** (transfer task):<br>- 2 random & 2 repeated blocks<br>**Day 16** (delayed retention)<br>- SRT: 6 repeated blocks<br>- Transfer: 2 repeated & 2 random blocks<br><br><u>Hand used:</u><br>Stroke: Ipsilesional hand<br>Control: Right hand	Mean difference in response time between block 17 (repeated) and block 18 (random) at end of acquisition phase on day 2	**Results:**<br>Aware = ?<br><br><u>Stroke:</u><br>Recall = 47 errors ± 10<br>[i.e., 73 correct responses]<br><br><u>Control:</u><br>Recall = 27 errors ± 9<br>[i.e., 93 correct responses]
Pohl et al. [78]<br><br>- Stroke group<br>- Control group	<u>Stroke:</u><br>- ≥ 60 years old<br>- > 6 months since stroke<br>- Stroke affecting AC<br>- Community-dwelling<br>- Right hand dominant<br>- MMSE > 17<br>- Able to sit independently<br>- No upper extremity impairment<br>- No uncorrected visual impairment<br>- No apraxia<br><br><u>Control:</u><br>- Same criteria + no neurological impairment	<u>Stroke:</u><br>- *N* (m/f) = 47 (29/18)<br>- Age (y) = 71 ± 6<br>- Months since stroke = 43 ± 61<br>- Stroke location = (sub)cortical-AC<br>- Lesion side (L/R) = ?<br>- MMSE = 27.5<br>- Motor functioning = ?<br>- General level of impairment Orpington:<br>- 18 patients: mild (< 3.2)<br>- 9 patients: moderate (3.2-5.2)<br>- 20 patients: ?<br><br><u>Control:</u><br>- *N* (m/f) = 36 (15/21)<br>- Age (y) = 73 ± 6<br>- MMSE = 28.6	SHM task<br><br><u>Procedure:</u><br>Block = 10 repetitions of 8-item sequence<br><br>**Day 1** (practice)<br>- Blocks 1 & 5-6: random<br>- Blocks 2-4 & 7-8: repeated<br>**Day 2** (retention)<br>- 2 repeated blocks<br><br><u>Hand used:</u><br>Stroke: ipsilesional hand<br>Control: matched to stroke group	Mean response (ms) time per block<br><br><u>Implicit motor learning:</u><br>Mean difference in response time between block 4 (repeated) and 5 (random) at end of practice	**Tests used:**<br>- Awareness (% of participants)<br>- Recall (# items)<br><br>**Results:**<br><u>Stroke:</u><br>Aware = 68%<br>Recall = 2.8 items ± 2.7<br><br><u>Control:</u><br>Aware = 75%<br>Recall = 2.4 items ± 1.8
Pohl et al. [72]<br><br>- Mild stroke group<br>- Moderate stroke group<br>- Control group	<u>Stroke:</u><br>- ≥ 50 years old<br>- 30-150 days since stroke<br>- No pre-existing disability<br>- Community dwelling	<u>Stroke (mild):</u><br>- *N* (m/f) = 22 (13/9)<br>- Age (y) = 72 ± 9<br>- Months since stroke = ?<br>- Stroke location =	SHM task<br><br><u>Procedure:</u><br>Block = 10 repetitions of 8-item sequence	Mean response time (ms) per block<br><br><u>Implicit motor learning:</u><br>Mean difference in response	**Tests used:**<br>- Awareness (% of participants)<br>- Recall (# items)<br><br>**Results:**

	- Right hand dominant<br>- MMSE > 23<br>- Able to sit independently<br>- Orpington score ≤ 5.2<br>- No uncorrected visual impairment<br>- No apraxia<br><br><u>Control:</u><br>- No history of neurologic impairment<br>- Right hand dominant	(sub)cortical-SupT<br>- Lesion side (L/R) = 13/9<br>- MMSE = 28.6 ± 2<br>- FAS (0-30) = 28 ± 2<br>- Orpington score < 3.2<br><u>Stroke (moderate):</u><br>- *N* (m/f) = 15 (5/10)<br>- Age (y) = 74 ± 9<br>- Months since stroke = ?<br>- Stroke location = (sub)cortical-SupT<br>- Lesion location (L/R) = 6/9<br>- MMSE = 26.6 ± 2.1<br>- FAS (0-30) = 27 ± 5<br>- Orpington: 3.2-5.2<br><br><u>Control:</u><br>- *N* (m/f) = 30 (5/25)<br>- Age (y) = 76 ± 7<br>- MMSE = 28.8 ± 1.3<br>- FAS (0-30) = 28 ± 1	**Day 1:**<br>8 blocks:<br>- Blocks 1-2 & 5: random<br>- Blocks 3-4 & 6: repeated<br><br><u>Hand used:</u><br>Stroke: ipsilesional hand<br>Control:<br>- 20 controls: right hand<br>- 20 controls: left hand	time between block 4 (repeated) and 5 (random)	<u>Stroke (mild):</u> Aware = 55%<br><u>Stroke (moderate):</u> Aware = 47%<br><u>Control:</u> Aware = 47%<br><br><u>All three groups combined:</u><br>Recall: 1.7 ± 2.2<br><br>"There was no difference between groups in the number of responses of the repeated sequence that could be recalled" (p. 251)
Rösser et al. [70]<br><br>- Stroke group (levodopa placebo condition)	<u>Stroke:</u><br>- 50-80 years old<br>- (Sub)cortical stroke<br>- > 1 year since stroke<br>- Initial MRC <2, but current MRC ≥ 4.5<br>- MMSE ≥ 27<br>- No untreated cardiac, metabolic, or psychiatric disease<br>- No drug (ab)use<br>- No hypersensitivity for levodopa/carbidopa	<u>Stroke:</u><br>- *N* (m/f) = 18 (13/5)<br>- Age (y) = 66 ± 7<br>- Months since stroke = 40 ± 25<br>- Stroke location = (sub)cortical-SupT<br>- Lesion side (L/R) = 11/7<br>- MMSE = 29.4 ± 0.6<br>- RMA-AS (0-15) = 12 ± 2	SRT task<br><br><u>Procedure:</u><br>Block = 500 keypresses with shorter and longer sequential elements intermixed with random ones (i.e., 85% repeated and 15% random per block)<br><br>**Session 1:**<br>2 blocks (with levodopa-placebo)<br><br><u>Hand used:</u><br>Contralesional hand	Mean response (ms) time for random and sequenced items per block<br><br><u>Implicit motor learning:</u><br>Mean difference in response time between random and sequenced items in the second block (for the placebo condition)	**Tests used:**<br>- Awareness (% of participants)<br><br>**Results:**<br>Aware: ?
Shin et al. [51]<br><br>- Stroke group<br>- Control group	<u>Stroke:</u><br>- Unilateral BG-stroke<br><br><u>Control:</u><br>- No neurologic impairment	<u>Stroke:</u><br>- *N* (m/f) = 4 (3/1)<br>- Age (y) = 65 ± 9<br>- Months since stroke = ?<br>- Stroke location =	SRT task<br><br><u>Procedure:</u><br>Block = 7 repetitions of 8-item sequence	Median response time (ms) per block<br><br><u>Implicit motor learning:</u><br>Mean difference in response	**Tests used:**<br>- Awareness (% of participants)<br>- Recall (# items)<br><br>**Results:**

	- Age-matched	Subcortical-BG<br>- Lesion side (L/R) = 2/2<br>- MMSE = 26.8 ± 3.9<br>- Fast-tapping task (interval in ms):<br>Contralesional hand = 240 ± 98<br>Ipsilesional hand = 203 ± 30<br><br><u>Control:</u><br>- *N* (m/f) = 7 (5/2)<br>- Age (y) = 68 ± 4<br>- MMSE = (all ≥ 29)<br>- Fast-tapping task (interval in ms):<br>Dominant hand = 171 ± 16	**Day 1**<br>7 practice blocks:<br>- Blocks 1-2: random, 3-7: repeated<br>3x4 post-test blocks<br>- Blocks 1&4: repeated, 2-3 either:<br>1) random stimuli location<br>2) random interstimulus interval<br>3) phase-shift interstimulus interval<br><br><u>Hand used:</u><br>Stroke: Both hands tested separately<br>Control: Dominant (right) hand	time between first repeated and subsequent random block in post-test no. 1 (random stimuli location; spatial learning test)[b]	<u>Stroke & Control</u><br>Aware: ?<br>Recall: all participants < 3<br><br>"None of the control participants or patients could correctly report parts of either sequence longer than two successive sequence elements." (p. 78)
Vakil et al. [55]<br><br>- Stroke group<br>- Control group	<u>Stroke:</u><br>- Isolated BG-lesions<br>- No previous head trauma, or neurological/endocrine disease<br>- No drug-use that could alter cognitive performance<br>- No dementia<br><br><u>Control:</u><br>- Age- & education-matched<br>- Right handed<br>- No neurological impairment	<u>Stroke:</u><br>- *N* (m/f) = 16 (11/5)<br>- Age (y) = 59 ± 11<br>- Months since stroke = 16<br>- Stroke location = Subcortical-BG<br>- Lesion side (L/R) = 5/11<br>- Years of education = 11 ± 3<br>- Motor functioning = ?<br><br><u>Control:</u><br>- *N* (m/f) = 16 (7/9)<br>- Age (y) = 58 ± 8<br>- Years of education = 12 ± 3<br>- Motor functioning = ?	SRT task<br><br><u>Procedure:</u><br>Block = 10 repetitions of 10-item sequence<br><br>**Day 1** (practice):<br>- Blocks 5: random<br>- Blocks 1-4: repeated<br>**Day 2** (retention): 1 repeated block<br><br><u>Hand used:</u><br>Stroke & Control: Middle and index finger of each hand	Median response time (ms) per block<br><br><u>Implicit motor learning:</u><br>Mean difference in response time between block 4 (repeated) and block 5 (random)	**Tests used:**<br>- Recall/Prediction (# items)<br>- Chance = 2.5<br><br>**Results:**<br><u>Stroke:</u><br>Recall = 6.1 ± 1.9<br><br><u>Control:</u><br>Recall = 5.4 ± 1.9

NB: AC = Anterior circulation; ARAT = Action Research Arm Test; BG = Basal ganglia; BBS = Berg Balance Scale; CB = Cerebellum; CBTT = Corsi block tapping test; CT = Continuous tracking task; DT = Dual-task; FAS = Florida Apraxia Screen; FMA-UE = Upper extremity subscale of Fügl-Meyer Assessment; ICARS = International Cooperative Ataxia Rating Scale (motor impairment scale); MCA = Middle cerebral artery; MMSE = Mini-Mental State Examination; MRC = Medical Research Council scale for muscle strength; PP (UL/UR/BL/BR) = Purdue Pegboard (unilateral left hand score/unilateral right hand score/bilateral left hand score/bilateral right hand score); RMA-AS = Rivermead Motor Assessment, arm section; SHM = Serial hand movement task SMC = Sensorimotor cortex; SRT = Serial reaction time task; ST = Single-

task; SupT = Supratentatorial; WAIS-R = Wechsler Adult Intelligence Scale – Revised; WM = working memory; # = number of;
[a] Two different random blocks were tested, Re and Rm. For the Re random blocks, each stimulus and transition between stimuli was of equal probability. For the Rm random blocks, stimulus (-transition) probability was the same as for the sequence learned in the practice blocks. We chose to only look at the difference in reaction times between the last Rm (and not Re) block and the final repeated sequence block, as this provides the most conservative measure of implicit motor learning.
[b] This test of spatial learning is actually the conventional test of implicit motor learning (i.e., difference in reaction time to random and sequenced stimuli)
