# Supplementary material for: Is Implicit Motor Learning Preserved after Stroke? A Systematic Review with Meta-Analysis
Source: PLoS One. 2016 Dec 16;11(12):e0166376. doi: 10.1371/journal.pone.0166376 (PMC5161313; doi:10.1371/journal.pone.0166376)
Supplement: S1 Text — Example of the search strategy for Medline. (DOCX) [file pone.0166376.s005.docx]

**S1 Text. Search Strategy.**

Example of the search strategy for Medline.

| **#1** | (Implicit*[tiab] OR procedural*[tiab] OR sequen*[tiab] OR unintentional*[tiab] OR incidental*[tiab] OR nondeclarative[tiab] OR non declarative[tiab] OR analogy[tiab] OR errorless[tiab] OR dual task[tiab] OR external focus[tiab] OR Implicit*[ot] OR procedural*[ot] OR sequen*[ot] OR unintentional*[ot] OR incidental*[ot] OR nondeclarative[ot] OR non declarative[ot] OR analogy[ot] OR errorless[ot] OR dual task[ot] OR external focus[ot]) |
| --- | --- |
| **#2** | ("Learning"[Mesh] OR Learn*[tiab] OR Learn*[ot]) |
| **#3** | (memory[tiab] OR knowledge[tiab] OR memory[ot] OR knowledge[ot]) |
| **#4** | ("Psychomotor Performance"[Mesh] OR Psychomotor*[tiab] OR Motor*[tiab] OR Task perform*[tiab] OR Task sequen*[tiab] OR Reaction time*[tiab] OR Psychomotor*[ot] OR Motor*[ot] OR Task perform*[ot] OR Task sequen*[ot] OR Reaction time*[ot])) |
| **#5** | ((("Stroke"[Mesh] OR cva[tiab] OR cvas[tiab] OR poststroke*[tiab] OR stroke*[tiab] OR apoplex*[tiab]) OR (brain*[tiab] OR cerebr*[tiab] OR cerebell*[tiab] OR intracran*[tiab] OR intracerebral*[tiab] OR vertebrobasilar*[tiab]) AND vascular*[tiab] AND (disease[tiab] OR diseases[tiab] OR accident*[tiab] OR disorder*[tiab])) OR (cerebrovascular*[tiab] AND (disease[tiab] OR diseases[tiab] OR accident*[tiab] OR disorder*[tiab])) OR ((brain*[tiab] OR cerebr*[tiab] OR cerebell*[tiab] OR intracran*[tiab] OR intracerebral*[tiab] OR vertebrobasilar*[tiab]) AND (haemorrhag*[tiab] OR hemorrhag*[tiab] OR ischemi*[tiab] OR ischaemi*[tiab] OR infarct*[tiab] OR haematoma*[tiab] OR hematoma*[tiab] OR bleed*[tiab])) OR ("Hemiplegia"[Mesh] OR "Paresis"[Mesh] OR hemipleg*[tiab] OR hemipar*[tiab] OR paresis[tiab] OR paretic[tiab])) OR ("Brain Injuries"[Mesh] OR brain injur*[tiab] OR brain trauma*[tiab] OR brain lesion*[tiab] OR brain laceration*[tiab] OR brain contusion*[tiab] OR brain damage[tiab] OR concussion*[tiab] OR cerebral injur*[tiab] OR cerebral trauma*[tiab] OR cerebral lesion*[tiab] OR cerebral laceration*[tiab] OR cerebral contusion*[tiab] OR cerebral damage[tiab] OR repeated head trauma[tiab] OR repetitive head trauma[tiab] OR traumatic encephalopath*[tiab] OR tbi[tiab] OR tbis[tiab] OR ctbi-b[tiab] OR contrecoup[tiab] OR post-concussi*[tiab] OR postconcussi*[tiab] OR post-trauma*[tiab] OR posttrauma*[tiab] OR traumatic brain*[tiab] OR traumatic midbrain*[tiab] OR traumatic cerebellar*[tiab] OR traumatic intracerebellar*[tiab] OR traumatic intra-cerebellar*[tiab] OR traumatic cerebral*[tiab] OR traumatic intracerebral*[tiab] OR traumatic intra-cerebral*[tiab] OR axonal injur*[tiab] OR dai[tiab] OR dais[tiab] OR traumatic epileps*[tiab] OR impact seizure*[tiab] OR concussive convulsion*[tiab] OR commotio cerebri[tiab] OR ((prefrontal[tiab] OR frontal[tiab] OR basal ganglia[tiab] OR striat*[tiab] OR parietal[tiab] OR cerebel*[tiab]) AND (lesion*[tiab] OR damag*[tiab])))) |
| **#6** | ((#1 AND (#2 OR #3)) AND # 4 AND 5#) NOT ("Animals"[Mesh] NOT "Humans"[Mesh]) |
