# Supplementary material for: Is Implicit Motor Learning Preserved after Stroke? A Systematic Review with Meta-Analysis
Source: PLoS One. 2016 Dec 16;11(12):e0166376. doi: 10.1371/journal.pone.0166376 (PMC5161313; doi:10.1371/journal.pone.0166376)
Supplement: S2 Text — Appendices A-C with Modified Newcastle Ottawa Scales. The three different Newcastle Ottawa Scales used to assess studies’ risk of bias for each of the three research questions. Of note, for each NOS-scale the items on performance bias rated studies’ quality on their success of blinding participants (i.e., the amount of explicit knowledge that participants gained with practice). In the NOS-scales used in this study, these items were given extra weight (i.e., 2 points could be scored per item, instead of 1), as it is the hallmark of implicit motor learning that learners do not gain explicit movement-related knowledge. Studies in which learners gained considerable explicit knowledge run the risk of having measured a more explicit form of motor learning. (DOCX) [file pone.0166376.s006.docx]

**S2 Text. Appendices A-C with Modified Newcastle Ottawa Scales**

**Appendix A.**

**NOS - version 1: Tool to assess risk of bias of studies for research question 1: Can patients with stroke learn motor tasks implicitly?**

**Selection**

1) Representativeness of patient group

*- One star was awarded when in- and exclusion criteria and patient characteristics were described (i.e., diagnosis, lesion location, time post-stroke, and motor and cognitive abilities)*

2) Selection of patient group

*- Studies that provided a detailed description of the recruitment of patients were awarded a star. (Where were patients included, how many patients were screened, and how many of them eventually participated?)*

**Performance bias**

3) Blinding of patients (check of explicit knowledge)
- *Stars were awarded if patients’ explicit knowledge of the learned motor task was comprehensively tested and reported. Thus, for SRT-type tasks, one star was awarded if at least a recognition or recall test was administered. Optionally, two stars could be awarded if both these tests were used. For complex motor tasks, one star was awarded if the number of verbal movement related rules was assessed*

4) Blinding of patients (explicit knowledge results)

*- Stars were awarded if blinding of participants’ was proven successful. That is, if the results of the* *explicit knowledge tests indicated that patients did not accumulate explicit knowledge. Thus, for SRT-type tasks, two stars were awarded if recognition/recall scores were not above chance levels. For complex motor tasks, two stars were awarded if significantly fewer verbal movement related rules were reported by the implicit group than by the explicit group*

**Outcome**

5) Was follow-up long enough for outcomes to occur?

*- One star was awarded if a separate retention test was included (>24 hours post-practice)*

6) Follow-up adequacy

*- One star was awarded if ≤ 10% of the subjects was lost to follow-up*

**Appendix B.**

**NOS-version 2: Tool to assess risk of bias of studies for research question 2: Is implicit motor learning of patients impaired compared to healthy peers?**

**Selection**

1) Representativeness of patient group

*- One star was awarded when in- and exclusion criteria and patient characteristics were described (i.e., diagnosis, lesion location, time post-stroke, and motor and cognitive abilities)*

2) Selection of patient group

*- Studies that provided a detailed description of the recruitment of patients were awarded a star. (Where were patients included, how many patients were screened, and how many of them eventually participated?)*

3) Selection of control group
*- Studies that selected control subjects from the same community as the stroke patient group were awarded a star*

**Performance bias**

4) Blinding of participants (check of explicit knowledge)
- *Stars were awarded if patients’ explicit knowledge of the learned motor task was comprehensively tested and reported. Thus, for SRT-type tasks, one star was awarded if at least a recognition or recall test was administered. Optionally, two stars could be awarded if both these tests were used. For complex motor tasks, one star was awarded if the number of verbal movement related rules was assessed*

5) Blinding of participants (explicit knowledge results)

*- Stars were awarded if blinding of participants’ was proven successful. That is, if the results of the* *explicit knowledge tests indicated that patients did not accumulate explicit knowledge. Thus, for SRT-type tasks, two stars were awarded if recognition/recall scores were not above chance levels. For complex motor tasks, two stars were awarded if significantly fewer verbal movement related rules were reported by the implicit group than by the explicit group*

**Comparability**

6) Comparability of groups (1)

*- One star was awarded when possible confounders were reported. At least the following information should be obtained: age, motor functioning, and cognitive functioning/education level*

7) Comparability of groups (2)

- *One star was awarded when groups were matched with regard to possible confounders or if confounders were statistically corrected for. At least 2 of the following 3 confounders should be taken into account: Age, motor functioning, and cognitive functioning/education level*

8) Comparability of groups (3)

- *One star was awarded when the amount of explicit knowledge was similar for patient and control groups. Alternatively, one star was awarded if follow-up analyses revealed that differences in explicit knowledge could not explain differences in learning between groups*

**Outcome**

9) Was follow-up long enough for outcomes to occur?

*- One star was awarded if a separate retention test was included (>24 hours post-practice)*

10) Follow-up adequacy

*- One star was awarded if ≤ 10% of the subjects was lost to follow-up*

**Appendix C.**

**NOS-version 3: Tool to assess risk of bias of studies for research question 3: Is implicit motor learning more or less impaired than explicit motor learning following stroke?**

**Selection**

1) Representativeness of patient group

*- One star was awarded when in- and exclusion criteria and patient characteristics were described (i.e., diagnosis, lesion location, time post-stroke, and motor and cognitive abilities)*

2) Selection of implicit stroke group

*- Studies that provided a detailed description of the recruitment of patients were awarded a star. (Where were patients included, how many patients were screened, and how many of them eventually participated?)*

3) Selection of explicit stroke group
*- Studies that selected patients of the explicit group from the same community as those from the implicit group were awarded a star*

**Performance bias**

4) Blinding of participants (check of explicit knowledge)
*- Stars were awarded if patients’ explicit knowledge of the learned motor task was comprehensively tested and reported. Thus, for SRT-type tasks, one star was awarded if at least a recognition or recall test was administered. Optionally, two stars could be awarded if both these tests were used. For complex motor tasks, one star was awarded if the number of verbal movement related rules was assessed*

5) Blinding of participants (explicit knowledge results)

- *Stars were awarded if blinding of participants’ was proven successful. That is, if the results of the* *explicit knowledge tests indicated that patients did not accumulate explicit knowledge. Thus, for SRT-type tasks, two stars were awarded if recognition/recall scores were not above chance levels. For complex motor tasks, two stars were awarded if significantly fewer verbal movement related rules were reported by the implicit group than by the explicit group*

**Comparability**

6) Comparability of groups (1)

*- One star was awarded when possible confounders were reported. At least the following information should be obtained: age, motor functioning, cognitive functioning/education level, and lesion location*

7) Comparability of groups (2)

- *One star was awarded when groups were matched with regard to possible confounders or if confounders were statistically corrected for. Besides lesion location and time since stroke, at least 2 of the following 3 confounders should be taken into account: Age, motor functioning, and cognitive functioning/education level*

8) Comparability of groups (3)

- *One star was awarded when the explicit group gained more explicit knowledge than the implicit group*

**Outcome**

9) Was follow-up long enough for outcomes to occur?

*- One star was awarded if a separate retention test was included (>24 hours post-practice)*

10) Follow-up adequacy

*- One star was awarded if ≤ 10% of the subjects was lost to follow-up*
